# Effects of reversible SERCA inhibition on catecholamine exocytosis and intracellular [Ca^2+^] signaling in chromaffin cells from normotensive Wistar Kyoto rats and spontaneously hypertensive rats

**DOI:** 10.1007/s00424-023-02859-z

**Published:** 2023-09-29

**Authors:** Oscar J. Parada-Parra, Arturo Hernández-Cruz

**Affiliations:** Departamento Neurociencia Cognitiva, and Laboratorio Nacional de Canalopatías, Instituto de Fisiología Celular, Universidad Nacional Autónoma de México, Circuito de La Investigación Científica S/N, Ciudad Universitaria, Mexico City CDMX, C.P. 04510 México

**Keywords:** Catecholamine secretion, Intracellular calcium, Adrenal medulla, Chromaffin cell, Hypertension, Ryanodine, Cyclopiazonic acid, WKY rats, SHR rats

## Abstract

Intracellular Ca^2+^ ([Ca^2+^]i) signaling and catecholamine (CA) exocytosis from adrenal chromaffin cells (CCs) differ between mammalian species. These differences partly result from the different contributions of Ca^2+^-induced Ca^2+^-release (CICR) from internal stores, which boosts intracellular Ca^2+^ signals. Transient inhibition of the sarcoendoplasmic reticulum (SERCA) Ca^2+^ pump with cyclopiazonic acid (CPA) reduces CICR. Recently, Martínez-Ramírez et al. found that CPA had contrasting effects on catecholamine secretion and intracellular Ca^2+^ signals in mouse and bovine CCs, where it *enhanced* and *inhibited* exocytosis, respectively. After CPA withdrawal, exocytosis diminished in mouse CCs and increased in bovine CCs. These differences can be explained if mouse CCs have weak CICR and strong Ca^2+^ uptake, and the reverse is true for bovine CCs. Surprisingly, CPA slightly reduced the amplitude of Ca^2+^ signals in both mouse and bovine CCs. Here we examined the effects of CPA on stimulated CA exocytosis and Ca^2+^ signaling in rat CCs and investigated if it alters differently the responses of CCs from normotensive (WKY) or hypertensive (SHR) rats, which differ in the gain of CICR. Our results demonstrate that CPA application strongly inhibits voltage-gated exocytosis and Ca^2+^ transients in rat CCs, regardless of strain (SHR or WKY). Thus, despite the greater phylogenetic distance from the most recent common ancestors, suppression of endoplasmic reticulum (ER) Ca^2+^ uptake through CPA inhibits the CA secretion in rat CCs more similarly to bovine than mouse CCs, unveiling divergent evolutionary relationships in the mechanism of CA exocytosis of CCs between rodents. Agents that inhibit the SERCA pump, such as CPA, suppress catecholamine secretion equally well in WKY and SHR CCs and are not potential therapeutic agents for hypertension. Rat CCs display Ca^2+^ signals of varying widths. Some even show early and late Ca^2+^ components. Narrowing the Ca^2+^ transients by CPA and ryanodine suggests that the late component is mainly due to CICR. Simultaneous recordings of Ca^2+^ signaling and amperometry in CCs revealed the existence of a robust and predictable correlation between the kinetics of the whole-cell intracellular Ca^2+^ signal and the rate of exocytosis at the single-cell level.

## Introduction

Historically, bovine and rat adrenal chromaffin cells (CCs) have been extensively used for neurobiology research [10, 23]; while mouse CCs have been used comparatively less. Mice and rats have long served as the preferred species for biomedical research due to their anatomical, physiological, and genetic similarity to humans. The advantages of rodents include their small size, ease of maintenance, short life cycle, and abundant genetic resources [8]. The mouse model has received significant interest because of an array of nearly 60,000 items in the catalog of the MMRRC, a resource of mutant mice available for biomedical research [6]. Naturally, bovine tissues do not arouse the same interest.

A series of comparative functional studies between mice and bovine CCs over the last 20 years have revealed significant differences in the mechanisms involved in regulating the dynamics of [Ca^2+^]i and catecholamine exocytosis. These functional disparities between species probably represent an evolutionary adaptation of autonomic stress responses. A similar comparison between mice and rats is lacking, perhaps because of the assumption that these species have a closer phylogenetic relationship.

### Role of the mitochondria and the endoplasmic reticulum on intracellular calcium signaling and homeostasis

Intracellular Ca^2+^ signals result from the sum of Ca^2+^ influx through the plasma membrane by way of Ca^2+^-permeant channels and transporters and Ca^2+^ release from intracellular Ca^2+^ stores. The mitochondria and the endoplasmic reticulum (ER) are the main intracellular Ca^2+^ stores. The mitochondrial Ca^2+^ uniporter (MCU) allows Ca^2+^ influx into the mitochondria. This cation movement is driven by the strongly negative membrane potential across the inner mitochondrial membrane (about -180 mV). When the [Ca^2+^]i near the MCU is > 1 µM, the channel opens and allows Ca^2+^ influx into the matrix. Mitochondrial efflux is primarily attributed to the Na^+^-Ca^2+^-Li^+^ exchanger (NCLX). Mitochondrial [Na^+^] is lower than the cytosolic one, and this gradient is the driving force that the NCLX utilizes to extrude Ca^2+^. The NCLX usually functions as a Ca^2+^ extrusion pathway, but it can lead to Ca^2+^ entry when the mitochondrial membrane potential dissipates and the Na^+^ gradient reverses [13, 31].

Ca^2+^ uptake is also mediated by the sarco/endoplasmic reticulum Ca^2+^ ATPase (SERCA), which exerts a buffering effect on [Ca^2+^]i, contributing to the maintenance of nanomolar resting [Ca^2+^]i. SERCA activity increases when the Ca^2+^ store is depleted, allowing the reuptake of cytosolic Ca^2+^. Rapid release of Ca^2+^ from the ER, required for many specific cell functions, is mediated mainly by two specialized intracellular Ca^2+^ channels from the ER membrane: InsP_3_ receptors and ryanodine receptors. Once G-proteins activate, phospholipase C acts on phosphatidylinositol 4,5- bisphosphate [PtdIns(4,5)P_2_] and releases InsP_3_ from the plasma membrane into the cytosol. InsP_3_ binds to its receptors (InsP_3_Rs), and Ca^2+^ is released. On the other hand, by binding Ca^2+^ on their cytoplasmic face, ryanodine receptors (RyRs) also allow Ca^2+^ release from the ER. In both cases, the Ca^2+^ rise triggers a self-sustained amplification cycle that is the basis for the so-called Ca^2+^-induced Ca^2+^ release (CICR) [29]. Depending on its Ca^2+^ content, the ER can attenuate or potentiate intracellular Ca^2+^ signals. When depleted, the ER may act as a Ca^2+^ sink. Conversely, when it is full of Ca^2+^, it may serve as a Ca^2+^ source, thus amplifying Ca^2+^ signals by CICR [7, 14].

### Stimulus-induced [Ca^2+^]i rise and secretory response in mice and bovine chromaffin cells

The dynamics of [Ca^2+^]i changes and exocytosis triggered by membrane depolarization are quite different between bovine and mouse CCs [2]. Without pharmacological interventions, stimulus-induced catecholamine secretion is similar in both species, but [Ca^2+^]i signals in bovine CCs are more robust and longer-lasting than in mouse CCs. Application of the protonophores CCCP/ FCCP, which dissipate the inner mitochondrial membrane potential, should stop the mitochondrial Ca^2+^ uptake by the MCU. With less Ca^2+^ buffering, [Ca^2+^]i signals should be potentiated. Nonetheless, in CCCP-treated mouse CCs, the secretory responses diminished by 51%, and the [Ca^2+^]i signal decreased by 48%, probably because the NCLX operates in the reverse mode in depolarized mitochondria [2]. Conversely, in CCCP-treated bovine CCs, the secretory response increased, as well as the amplitude and time course of the evoked [Ca^2+^]i rise [17]. These disparities in intracellular Ca^2+^ handling between species suggest that mitochondria from CCs play different roles in controlling [Ca^2+^]i and exocytosis.

Martínez-Ramírez et al. recently compared the effects of acute, reversible inhibition of SERCA with cyclopiazonic acid (CPA) on the exocytotic responses and [Ca^2+^]i transients elicited by brief applications of ACh in mouse and bovine CCs [25]. During CPA application, ACh-induced exocytosis increased slightly in mouse CCs while inhibited in bovine CC. On the other hand, CPA reduced slightly ACh-induced [Ca^2+^]i signals in both cell types. How can the inhibition of ER Ca^2+^ uptake by CPA affect ACh-elicited CA exocytosis differently in mouse and bovine CCs? This discrepancy could result from differences in the ER Ca^2+^ handling between CCs from these unrelated species. An interpretation of these results is that the primary role of the ER in mouse CCs is Ca^2+^ uptake. Therefore, these cells display strong Ca^2+^ buffering and weak CICR under steady-state conditions. The reverse would be valid for the role of the ER in bovine CCs [18].

The spontaneously hypertensive rats (SHR) [27] have been extensively used as an animal model of essential human hypertension [39]. Like humans, blood pressure in SHR rises with age: At 6–9 weeks, it is above 150 mmHg, and by 15 weeks of age, it plateaus at ~ 200 mmHg [21]. When stimulated by brief membrane depolarization, CCs from young adult SHRs produce a more robust overall catecholamine output than those from WKY, normotensive rats. These findings are consistent with larger depolarization-induced [Ca^2+^]i signals recorded in SHR CCs [26], attributed to a more substantial contribution of ER Ca^2+^-induced Ca^2+^ release in these cells [34]. Using single-cell amperometry and intracellular Ca^2+^-imaging, we aim to address the following questions:

1. How does the transient inhibition of ER Ca^2+^ uptake by CPA affect the depolarization-induced catecholamine exocytosis of CCs from SHR and WKY rats?
2. Does the effect of CPA on exocytosis of rat CCs resemble that observed in mouse CCs, or is it more like bovine CCs?
3. Does CPA application affect the voltage-gated Ca^2+^ transients in CCs from normotensive WKY and hypertensive SHR rats differently?

## Methods

### Animals

Animal procedures were performed following the guidelines of the Mexican Guide for the Care and Use of Laboratory Animals of the Secretary of Agriculture (SAGA RPA NOM-062-Z00–1999), and the Institutional Committee approved experimental protocols of Care and Use of Laboratory Animals (CICUAL-IFC: protocol # AHC24-141). Male Wistar Kyoto (WKY) normotensive and spontaneously hypertensive rats (SHR) 13–16 weeks old were used. The animals, originally purchased from Charles River Laboratories, Inc. (Wilmington, USA), have been bred in our animal facility for about two years. The animal facility is temperature-controlled (22 °C) and maintained on a 12-h light/dark cycle. Rats received standard laboratory chow and water ad libitum.

### Preparation of primary cultures of rat and bovine adrenal chromaffin cells

CC cultures were prepared as in [1]. Briefly, rats were anesthetized with ketamine-xylazine (80 mg/kg and 10 mg/kg) and sacrificed by decapitation. Adrenal glands were immersed in sterile ice-cold Krebs–Ringer Bicarbonate solution (KB) and continuously gassed with 95% O_2_–5% CO_2_. KB contains (in millimolar): 125 NaCl, 2.5 KCl, 2 CaCl_2_, 1 MgCl_2_, 26 NaHCO_3_, 1.25 NaH_2_PO_4_, and 10 glucose. Bovine adrenal glands were collected from a local slaughterhouse and transported to the lab in cold PBS on ice.

Rat chromaffin cells were isolated by digestion of the adrenal medulla according to [34] with some modifications [1] in Ca^2+^/Mg^2+^—free Hanks medium pH = 7.4 (Sigma-Aldrich, St Louis, MO, USA) containing 1.5 mg/ml collagenase type-1 (Worthington Biochem Corp., Lakewood, NJ, US) and 1 mg/ml DNase type-1 (Sigma-Aldrich) during 30–40 min at 37 °C at rest. For complete medulla dissociation, agitation by hand was done every 10 min, accompanied by mechanical abrasion of the medulla with the help of a glass Pasteur pipette of narrowing diameter at its tip. Our preparations were enriched in chromaffin cells. After digestion, CCs suspension was pelleted by centrifugation at 1000 rpm and then re-suspended in Dulbecco's modified Eagle's medium (DMEM) supplemented with 5% fetal calf serum, 5 μg/ml insulin (Sigma- Aldrich), and 1X antibiotic–antimycotic solution (Sigma-Aldrich A5955). Cells (5 × 10^6^ in 10 ml DMEM) were plated on poly-l-lysine loaded-18-mm circular glasses in a 12-well cell culture plate, kept in a water-saturated incubator at 37 °C, in a 5% CO_2_/95% air atmosphere, and used for 3–5 days afterward. Media were replaced by serum-free DMEM 24 h later and then every two days.

Bovine adrenal medullas were dissected and incubated in Ca^2+^/Mg^2+^-free Hanks medium pH = 7.4 (Sigma-Aldrich, St Louis, MO, USA) with 2 mg/ml collagenase type-1 (Worthington Biochem Corp., Lakewood, NJ, US) and 1.5 mg/ml DNase type-1 (Sigma-Aldrich) for 30–40 min at 37 °C. The resulting fragments were dissociated by gentle agitation by hand. The cell suspension was centrifuged, and the pellet was re-suspended in fresh Dulbecco's modified eagle medium (DMEM, Gibco, Life Technologies, Rockville, MD, USA) supplemented with 10% fetal bovine serum (FBS, Gibco), 5 μg/ml insulin (Sigma- Aldrich), and 1X antibiotic–antimycotic solution (Sigma-Aldrich A5955). Chromaffin cells were plated in 12-well cell culture plates (Costar Corning, New York, NY, USA) on poly-L-lysine (Sigma- Aldrich) treated number 1 round glass coverslips (Thomas Scientific, Swedesboro, NJ, USA). They remained in DMEM medium at 37 °C in a humidified atmosphere (95% air/5%CO_**2**_). Cells were used 24–48 h. after plating. All experiments were performed at room temperature (22 ± 2 °C).

### Amperometric recording of catecholamine exocytosis

Coverslips containing CCs were placed in an experimental chamber mounted on the stage of a Leica DMI4000B inverted microscope. This chamber was continuously perfused (2 ml/min) with Tyrode's solution containing (in mM): 137 NaCl, 5 KCl, 1 MgCl_2_, 2 CaCl_2_, 10 HEPES, 10 glucose, pH 7.4, titrated with NaOH. Exocytosis of catecholamines was detected with amperometry [37]. Home-made carbon-fiber microelectrodes were built as previously described [1]. Briefly, a carbon fiber 10 μm diameter was inserted into a white plastic pipette tip, thus insulating it with polypropylene. Then, it was heat-stretched until the resulting plastic cylinder wrapping the fiber reached single-cell dimensions in width. The carbon fiber was then exposed by cutting the plastic cylinder tip transversally with a blade and applying a brief heat pulse to project a portion of the carbon fiber that will contact the cell during recordings. Microelectrodes were connected to the amplifier's head stage via KCl 3 M solution. Before recordings, electrodes were calibrated with 50 μM adrenaline dissolved in standard Tyrode; only electrodes that yielded a current > 200 pA were used for experiments. Real-time catecholamine exocytosis was recorded with the microelectrode tip maintained at + 700 mV measured against an Ag/AgCl bath reference electrode and placed in close contact with the cell membrane with a micromanipulator (MPC-200, Sutter Instrument Company, Novato, CA, USA). Recordings were performed under voltage-clamp mode with an EPC-10 amplifier running the PatchMaster software (HEKA Electronic, Lambrecht, Germany). The amperometric currents were acquired at 4 kHz and low-pass filtered at 400 Hz. Amperometric recordings were performed at room temperature (22–24 °C). CCs were stimulated with eight 1-s -long depolarizing puffs of high K^+^ solution (containing 60 mM K^+^ and 2 mM Ca^2+^) separated by 30-s -long intervals. Solutions were applied through an electronic valve-controlled puffer tip placed at 200 μm from the cell under study. We recorded cells from left to right in the coverslip (bath solution running right to the left) so that a newly selected cell for recording had no contact with test solutions used previously. In this manner, 5–6 CCs could be recorded per coverslip**.** We tested the effects of briefly exposing the cells to 10 μM cyclopiazonic acid (CPA) or 10 μM ryanodine on the kinetic parameters of amperometric spikes (mean spike number and cumulative amperometric charge). One minute after the end of each recording, we applied a final puff of high K^+^ (60 mM; 1 s) to check for cell viability.

### Intracellular Ca^2+^ imaging

Cultures were incubated with 2 μM of the cell-permeable fluorescent Ca^2+^ indicator Fluo-4 AM (Molecular Probes, Life Technologies) dissolved in Tyrode at room temperature for 30 min. Then coverslip with cells was washed twice, placed in the bottom of a recording chamber attached to the stage of a Leica DMI4000B inverted microscope, and continuously perfused (2 mL/min) by gravity. A Leica Lamp system (EL6000) coupled with fiber optics was used for illumination. Excitation light was band passed (480/40 nm) with a filter cube (Leica L5) placed in the light path, and emitted fluorescence was band-passed (527/30 nm) before its reflection into the camera port. Fluorescence images were acquired at 60 ms exposure and 380-ms intervals with an oil-immersion Leica objective 40X and a cooled digital CCD camera (Andor Technology iXon 897). Illumination and acquisition were controlled with routines programmed in Micro-Manager software [12] version 1.4. At the beginning of the recording, cells remained undisturbed for about 5 min. Then, they were depolarized by a series of 8 consecutive 1-s -long puffs of high K^+^ solution delivered every 30 s by gravity through a perfusion system nearby. Separate lines, each controlled with a solenoid valve, converge in an outlet less than 1 cm long so that the dead volume is minimal**.** Pressure pulses were produced by an array of electronic valves from a custom-made controller device (OmniAlva devices; Mexico). Fluorescence image sequences (movies) of CCs were saved in multi-tiff format, processed, and analyzed with macros written in Image J version 1.38 (National Institutes of Health). Raw movies were converted to ΔF movies: ΔF = F(i) − F0, where F0 is the result of averaging the first five frames of the sequence, and F(i) represents any (i) fluorescence image of the set. Regions of interest (ROIs) were drawn around the contour of each cell that responded to high K^+^ with a fluorescence increase (SD ± 0.5). These ROIs were used for single-cell quantification of ΔF over time. We tested the effects of briefly exposing the cells to CPA (10 μM) or ryanodine (10 μM) on the half-width (HW) and normalized area under the curve (NA) of voltage-gated Ca^2+^ transients. In some experiments, the amperometric spikes and Ca^2+^ transients in response to the depolarizing pulses were obtained simultaneously from the same cell. CCs were incubated with Fluo-4 (2 µM) and placed on the inverted microscope stage described above. Then, a carbon-fiber microelectrode set at + 700 mV connected to the Patch clamp setup was placed in close contact with its cell membrane. The programs controlling the camera (Micro-Manager) and the acquiring of amperometric signals (Patchmaster) were turned on. After 1 min of recording, eight consecutive 1-s-long high K^+^ pulses were delivered alongside CPA during the 3^th^ through 5^th^ pulses as described previously. All experiments were performed at room temperature (22–24 °C).

### Analysis and statistics

Amperometric spike analysis was performed using the pulse program (HEKA, Lambrecht/Pfalz, Germany) and IgorPro software (Max Planck Institute, München, Germany), which includes Ricardo Borges's macro package that allows the analysis of single events [32]. The cumulative amperometric charge was calculated by integrating the amperometric current over time from each spike. The sum of spike areas in each depolarizing stimulus was taken as total secretion per stimulus. We used MATLAB (9.10.0 (R2021a), Natick, Massachusetts) to compute the first derivative of the Ca^2+^ signal and the cumulative charge of the amperometric response over time. For the kinetic analysis of voltage-gated Ca^2+^ transients, we calculated the first derivative of ΔF (t) using the command *diff X(t)*. This function calculates the difference between adjacent elements of *X* along time (*Y* = *[X(2)-X(1) X(3)-X(2) … X(m)-X(m-1)]*), returning the function *Y* = *diff X(t)* which is a vector of length *m-1* with the differences between adjacent elements of *X*. For the kinetic analysis of catecholamine exocytosis, we wrote a code in MATLAB® that summed every 0.5 s the charge from each amperometric spike identified with Borges's macro package [32]. The cumulative charge obtained was plotted as a function of time.

Graph drawings and mathematical analyses were performed using Microcal Origin, 6.0, and MATLAB R2021a. The mean, confidence interval, and standard error of the mean (SEM) values derived from the total secretion per stimulus for all the cells recorded from each rat strain were obtained and then pooled together for statistical comparison. We proceeded the same way for the half-width and the normalized area measured from Ca^2+^ transients elicited by high K^+^.

Before hypothesis testing, we checked for normality in the data set using the Shapiro–Wilk statistical criteria to decide which parametric/non-parametric statistics to use. When normality was corroborated, we used the analysis of variance (ANOVA); when not, we used a Mann–Whitney test to compare groups, as some data did not fit a normal distribution. *p < 0.05 was taken as the limit of significance, and **p < 0.01 and ***p < 0.001 were taken as additional statistical significance limits.

## Results

### Effects of repeated depolarization on CA exocytosis in CCs from normotensive WKY and hypertensive SHR rats

**Fig. 1:**
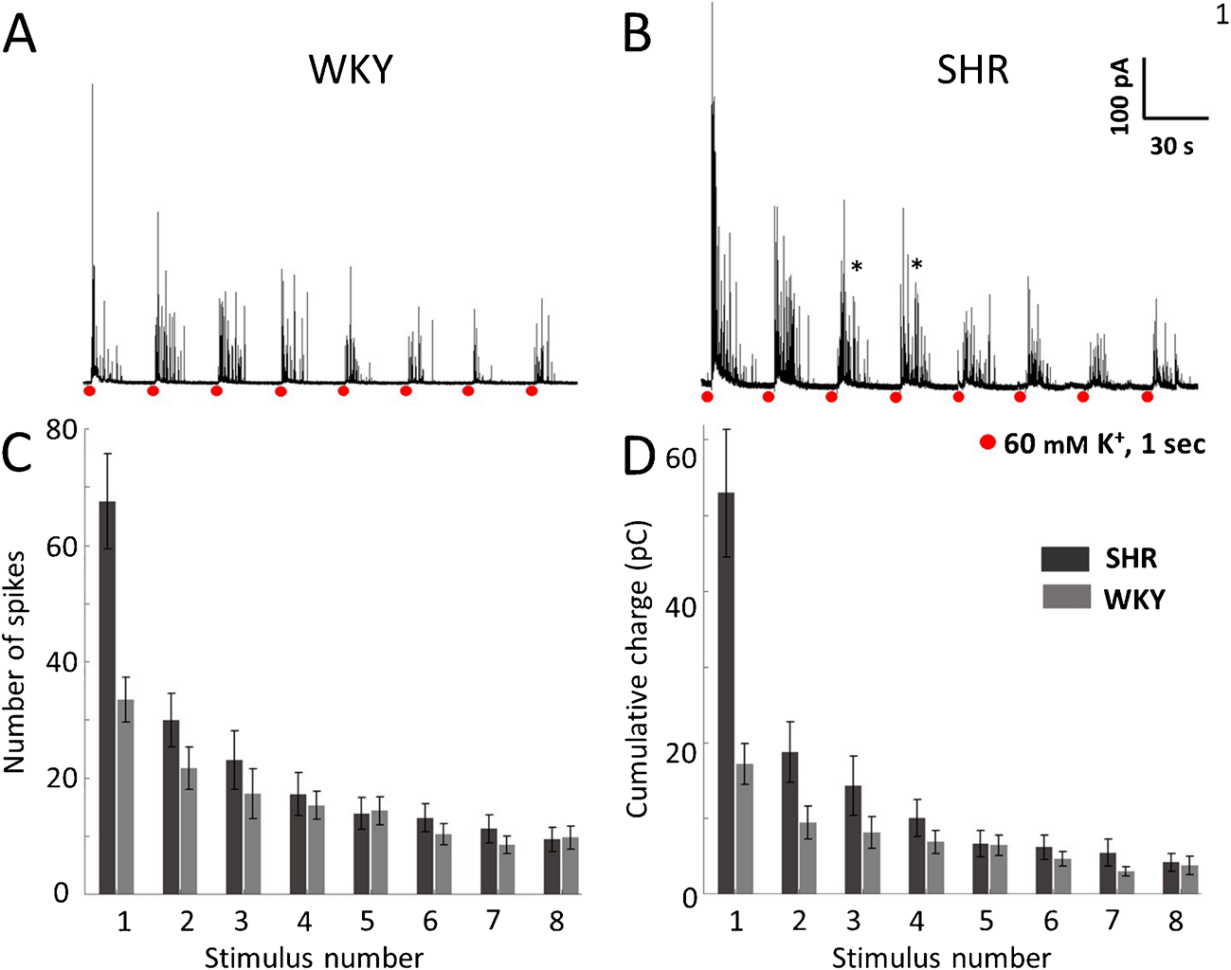
Amperometric recordings of voltage-gated exocytosis from untreated WKY and SHR CCs following recurrent depolarizing stimulation. Chromaffin cells from WKY rats and SHR were stimulated with eight pulses of high K^+^, 1-s in duration at intervals of 30 s. **A**, **B**: Representative amperometric recordings obtained from a WKY and a SHR CC under control conditions in response to depolarizing stimuli. The number of spikes (**C**) and the cumulative charge per stimulus (**D**) were quantified from these amperometric recordings in WKY (gray bars) and SHR CCs (black bars). *n* = 14 SHR CCs and 14 WKY CCs. See Table 1; *p < 0.05; **p < 0.01; ***p < 0.001. Asterisks indicate recordings with double bursts of amperometric spikes (see text)

To evaluate the effects of repeated transmembrane Ca^2+^ influx on CA exocytosis, we stimulated the cells with eight consecutive puffs of high K^+^ solution (60 mM, 1 s) at intervals of 30 s. We used depolarization rather than ACh to avoid complications due to the combined activation of nicotinic (nAChRs), muscarinic (mAChRs) receptors, and voltage-gated Ca^2+^ channels. Also, nicotinic receptor desensitization occurs upon repeated exposure to the agonist [11, 40]. Each pulse of high K^+^ opens voltage-gated Ca^2+^ channels (VGCC), allows Ca^2+^ entry, and produces an [Ca^2+^]i elevation that triggers exocytosis [9]. Figure 1A and B are representative amperometric recordings obtained from a WKY and a SHR CC under control conditions in response to depolarizing stimuli. As previously reported, SHR CCs produced more secretory events than WKY CCs after each stimulation; the increased CA secretion in SHR CCs likely results from a more significant contribution of the Ca^2+^-induced Ca^2+^-release mechanism [33, 34]. Also, the kinetic parameters of the amperometric spikes showed noticeable inter-strain differences (see Fig. 1C and D). The mean number of spikes and the cumulative charge over time elicited by the first stimulus of the series is about twofold (*p*-value < 0.01) and about threefold (*p*-value < 0.01) bigger, respectively, in SHR CCs compared to WKY CCs (Fig. 1C and D; see Table 1). Remarkably, the strength of the amperometric response diminishes considerably after the first stimulus in CCs from both strains (Fig. 1A and B). Accordingly, the number of spikes and the peak cumulative charge fell drasticall*y* between the first and the second stimuli, by 35% (*p* < 0.05) and 45% (*p* < 0.05), respectively, in WKY CCs and by 55% (*p* < 0.01) and 64% (*p* < 0.001), respectively in SHR CCs (Fig. 1C and D). By the 8^th^ stimulus, the number of spikes and the peak cumulative charge have fallen by 71% (*p* < 0.001) and 78% (*p* < 0.001) in WKY and by 86% (*p* < 0.001) and 92% (*p* < 0.001) in SHR CCs (Fig. 1C and D; see Discussion).

**Table 1:** Decay of exocytosis following repetitive stimulation of WKY and SHR CCs. The percentage of exocytosis produced by membrane depolarization from control, untreated CCs (stimulus 2 to 8) compared with the exocytosis triggered by the 1^st^ stimulus*.* (*n* = 14 WKY CCs and 14 SHR CCs, cultures from 6 rats of each strain)

Control		Percentage of exocytosis remaining compared to the 1^st^ stimulus						
		P2	P3	P4	P5	P6	P7	P8
Number of spikes per stimulus	WKY	64.6 ± 7.4	51.6 ± 10.9	45.6 ± 6.3	42.8 ± 16.7	30.7 ± 12.8	25.4 ± 8.5	28.9 ± 5.0
	SHR	44.3 ± 7.8	34.1 ± 9.1	25.5 ± 5.7	20.5 ± 4.4	19.4 ± 5.1	16.6 ± 4.2	14.0 ± 4.2
Peak cumulative charge	WKY	55.0 ± 9.5	47.4 ± 8.4	40.1 ± 11.1	37.5 ± 16.7	27.1 ± 9.3	17.4 ± 5.5	21.8 ± 5.4
	SHR	35.4 ± 7.6	26.9 ± 8.2	18.9 ± 6.6	12.5 ± 4.3	11.6 ± 4.4	10.3 ± 4.1	7.8 ± 3.1

### How does CPA affect the catecholamine exocytosis of CCs from SHR and WKY rats?

**Fig. 2:**
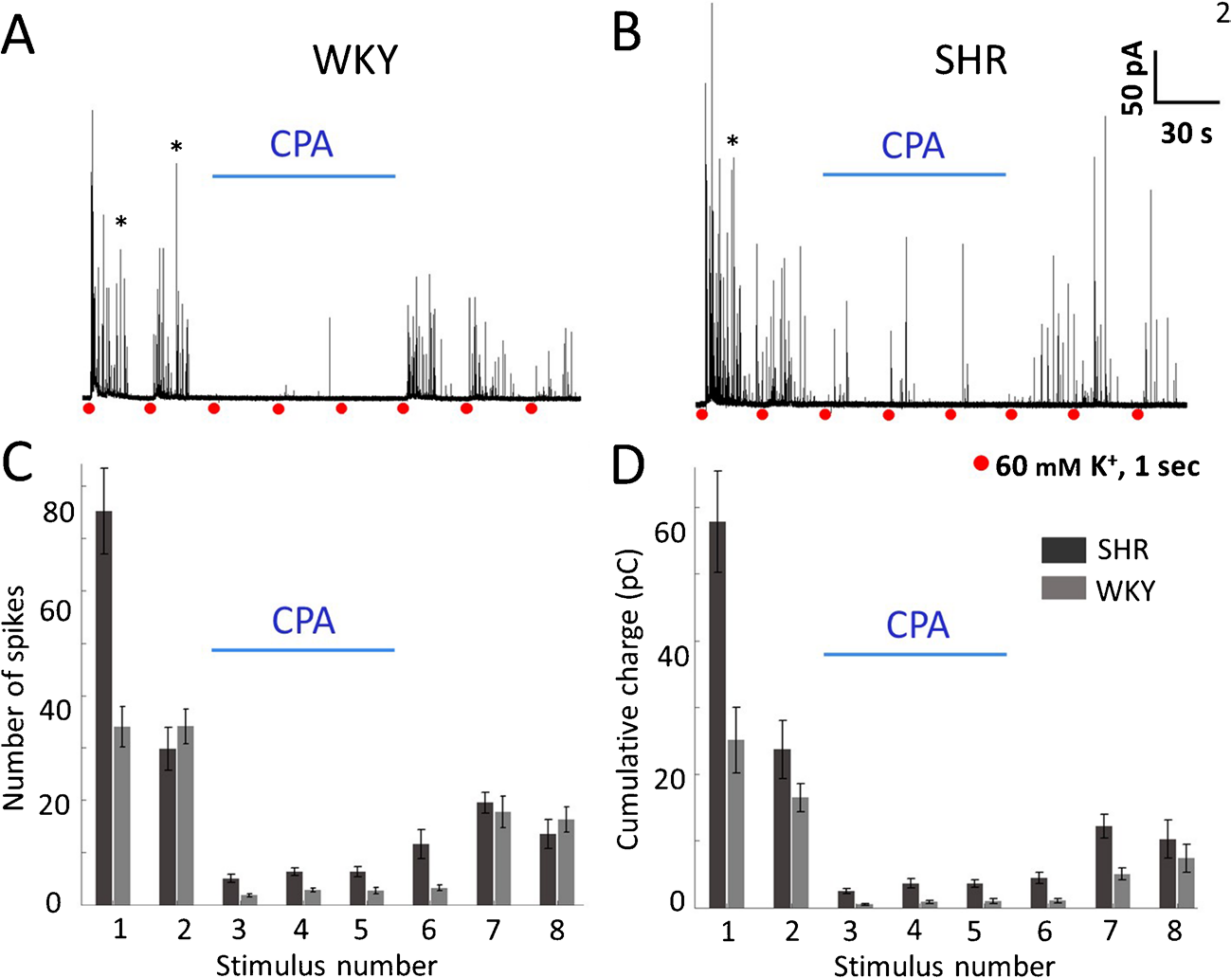
Effects of 10 µM CPA on recurrent stimulated exocytosis in WKY and SHR CCs. **A**, **B**. Amperometric spikes were recorded from individual WKY and SHR CCs. First, the cells were bathed in normal Tyrode while stimuli 1 and 2 were given. Then, Tyrode with CPA (10 µM) was applied for 90 s, while stimuli 3 through 5 were delivered. Lastly, CPA application was halted, and stimulation continued (stimuli 6 through 8). **C**, **D**: The number of amperometric spikes (**C**) and the cumulative charge per stimulus (**D**) were quantified in CCs from WKY rats (gray bars) and SHR (black bars) throughout the stimulation protocol. *n* = 27 SHR CCs and 27 WKY CCs *p < 0.05; **p < 0.01; ***p < 0.001. Asterisks indicate recordings with double bursts of amperometric spikes (see text)

Since the stimulated CA secretion in SHR CCs is greater because of the more significant contribution of Ca^2+^-induced Ca^2+^-release from intracellular stores [34], we speculated that CPA inhibition of ER Ca^2+^ uptake should have more impact on the amperometric responses of SHR than those of WKY CCs. Cells were first bathed in normal Tyrode while stimuli 1 and 2 were given. Then, CPA (10 µM) was applied for 90 s, while stimuli 3 through 5 were delivered. Lastly, CPA application was halted, and stimulation continued (stimuli 6 through 8). CPA almost entirely abolished the production of amperometric spikes in response to depolarizing stimuli in both strains (Fig. 2A and B)*.* CPA effects began rapidly and continued while present (stimulus 3–5). After CPA was withdrawn, the amperometric responses recovered readily but incompletely (Figs. 2A, B, C and D; stimuli 6–8). The quantitative impact of CPA and its withdrawal on the number of spikes and the cumulative charge of WKY and SHR CCs are summarized in Fig. 2C and D, and Table 2. The percentage of inhibition of the number of spikes and the cumulative charge (pulse #3 *versus* pulse #2) was 94% (*p* < 0.001) and 96% (*p* < 0.001) in WKY and 83% (*p* < 0.01) and 90% (*p* < 0.001) in SHR CCs. The incomplete recovery after CPA may be due, in part, to the gradual weakening of exocytosis observed in untreated CCs during recurrent stimulation (Fig. 1A, B).

**Table 2:** Inhibition of exocytosis by CPA in WKY and SHR CCs. The percentage of remaining exocytosis during CPA application (depolarizing stimuli 3 to 5) is compared to the exocytosis triggered after CPA washout (stimuli 6 to 8), (*n* = 27 WKY CCs and 27 SHR CCs, cultures from 6 rats from each strain)

CPA		Percentage of exocytosis remaining compared to the 2^nd^ stimulus					
		CPA			WASH		
		P3	P4	P5	P6	P7	P8
Number of spikes per stimulus	WKY	5.6 ± 2.6	8.5 ± 7.5	8.2 ± 6.9	9.7 ± 16.2	51.9 ± 16.4	47.8 ± 15.1
	SHR	17.1 ± 3.0	21.5 ± 3.4	21.5 ± 3.6	38.9 ± 9.8	65.4 ± 15.6	45.3 ± 9.0
Peak cumulative charge	WKY	3.4 ± 4.3	5.6 ± 7.7	6.2 ± 5.1	6.6 ± 21.6	31.0 ± 19.0	45.0 ± 16.7
	SHR	10.7 ± 2.9	15.6 ± 2.5	15.5 ± 3.6	18.9 ± 12.0	51.5 ± 14.4	43.3 ± 12.0

### Does the effect of CPA on rat CCs exocytosis resemble that observed in mouse CCs, or is it more similar to bovine CCs?

In mouse CCs, exocytosis increased slightly during CPA application with no significant effects after its withdrawal. Conversely, in bovine CCs, CPA inhibited considerably exocytotic responses and showed rapid recovery and rebound upon its removal [25]. Based on our earlier data [33, 34], we speculated that in SHR CCs, where Ca^2+^ release from intracellular stores is robust, CPA should significantly inhibit the amperometric responses (like in bovine CCs). Conversely, in WKY CCs, where Ca^2+^ release from intracellular stores is weak, CPA should affect less the amperometric responses (like in mouse CCs). The results shown in Fig. 2 demonstrate that the effect of CPA on the exocytosis of rat CCs resembles that observed in bovine CCs regardless of the strain (see Discussion).

### Effects of recurrent depolarization on Ca^2+^-rises of CCs from normotensive WKY rats and hypertensive SHR under control conditions

An intriguing result from the study by Martínez-Ramírez et al. [25] is that CPA strongly inhibited ACh-induced CA secretion in bovine CCs. In contrast, the amplitude of the ACh-induced Ca^2+^ transients diminished only slightly with CPA, both in mice and bovine CCs [25]. Here we used the low-affinity Ca^2+^ indicator Fluo-4 to examine more reliably the kinetic characteristics of [Ca^2+^]i signals. On the other hand, our stimulation protocol (1-s-long depolarizing stimuli separated by 30-s intervals) caused a considerable decline in the amperometric responses over time (see Fig. 1C, D). Thus we wondered if the underlying [Ca^2+^]i signals produced in response to repeated depolarizing stimuli also declined or remained stable.

**Fig. 3:**
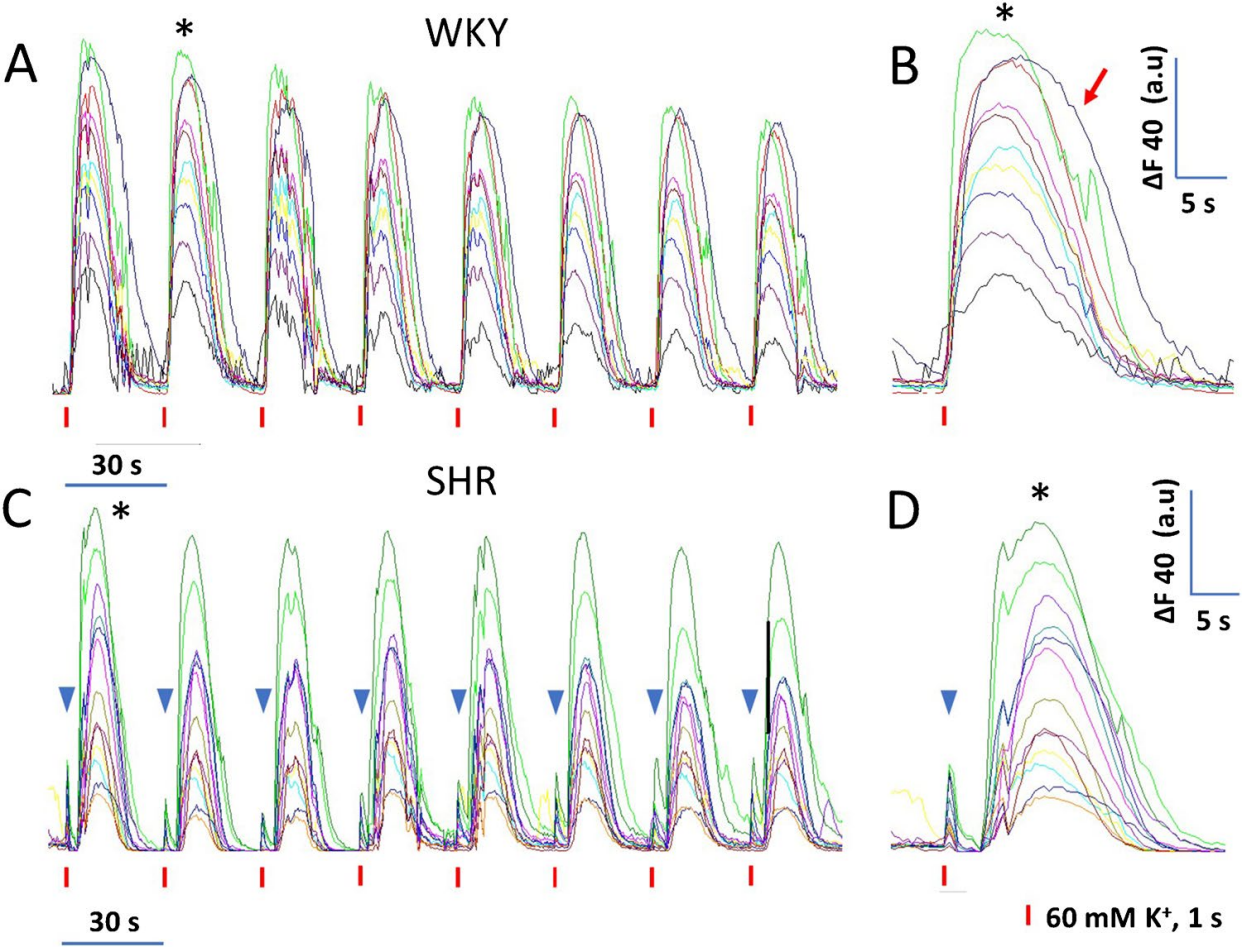
Voltage-gated intracellular Ca^2+^ signals recorded from untreated WKY and SHR CCs. **A**: Ten CCs from a WKY rat were stimulated with eight pulses of high K^+^, 1-s in duration at 30-s intervals. The recorded Ca^2+^ signals are shown superimposed in different colors. **B**. The Ca^2+^ transients elicited by the second stimulus in A (asterisk) are displayed with an expanded time scale. **C**. Ca^2+^ transients were recorded from a group of 13 SHR CC. Most of these CCs show early and late Ca^2+^ transient components. Blue triangles indicate each early component. The Ca^2+^ transients, elicited by the first stimulus in C (asterisk), are shown in **D** with an expanded time scale. The early and late components can be easily identified

Figure 3 exemplifies the effect of recurrent stimulation on Ca^2+^ transients recorded from untreated WKY and SHR CCs. Remarkably, the peak amplitude of these [Ca^2+^]i transients only declines slightly over time. At lower stimulation frequencies, the rate of decline was even less (data not shown). These examples illustrate the diversity of Ca^2+^ transient kinetics displayed. We did not use the peak amplitude to compare the properties of the Ca^2+^ transients because this parameter alone does not accurately represent Ca^2+^ mobilization triggered by the stimulus, and the recordings sometimes have two peaks (see below). Instead, we chose the *half-width* (HW) and the *normalized area* (NA) under the Ca^2+^ transients.

**Fig. 4:**
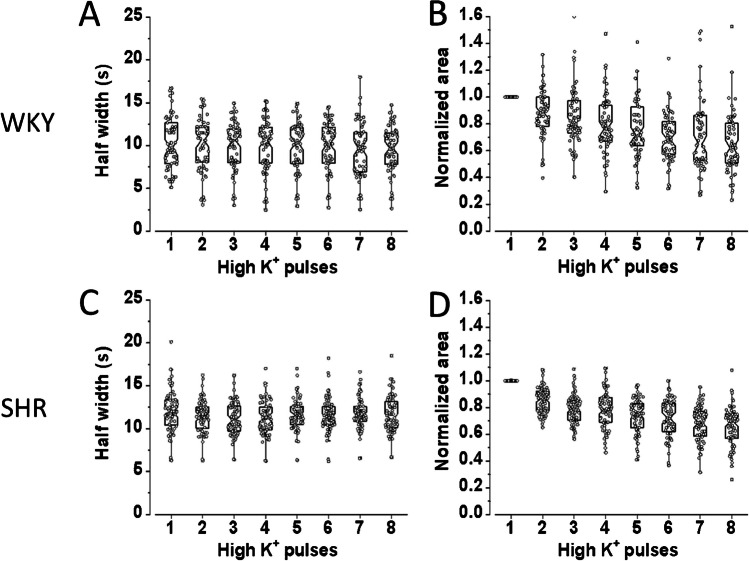
Changes in the half-width and normalized area of the Ca^2+^ transients from untreated WKY and SHR CCs that were stimulated recurrently. CCs from WKY and SHR were stimulated with eight pulses of high K^+^, 1-s in duration at 30-s intervals. The half-width and the normalized area characterize the kinetics of the Ca^2+^ transients from individual CCs.** A **and **C**: Hybrid plots showing the evolution of the half-width of the Ca^2+^ transients during recurrent depolarizing stimuli in WKY and SHR, respectively. **B** and **D**: Hybrid plots showing the development of the normalized area of the Ca^2+^ transients during recurrent depolarizing impulses in WKY and SHR, respectively. The half-width of the CCs Ca^2+^ transients remains relatively stable during recurrent stimulation. Conversely, the normalized area declines gradually after eight stimuli (see details in Tables 3 and 4). **A** and** B** *n* = 65 CCs; C and D: *n* = 68 CCs

As seen in Fig. 4A and C, recurrent stimulation of untreated CCs causes the half-width to shorten from 9.9 ± 0.4 s (second stimulus) to 9.5 ± 0.3 s (eighth stimulus; *n* = 65) and from 11.6 ± 0.2 s to 11.4 ± 0.3 s (*n* = 68), in WKY and SHR CCs, respectively. At the same time, the normalized area also diminishes from 89.0% ± 2.5 (second stimulus) to 65.1% ± 2.7 (eighth stimulus; *n* = 65) and from 85.1% ± 1.2 (second stimulus) to 65.9% ± 1.8 (eighth stimulus; *n* = 68) in WKY and SHR CCs, respectively (Fig. 4B and D). These data are summarized in Table 3. Thus, in contrast with the amperometric responses, which dropped drasticall*y* between the first and the second stimuli, the half-width of the Ca^2+^ transients remains relatively stable during recurrent stimulation (Fig. 4A and C). Conversely, the normalized area declines gradually, reaching 23.9% (WKY) and 19.2% (SHR) decay after eight stimuli (Fig. 4B and D).

**Table 3:** Decay of Ca^2+^ signals during repetitive stimulation in control, untreated WKY, and SHR CCs. The half-width and normalized area under the curve of the Ca^2+^ signals produced by membrane depolarization (stimulus 2 to 8) were compared to the Ca^2+^ signals triggered by the 1^st^ stimulus*.* (*n* = 65 WKY CCs and 68 SHR CCs, cultures from 3 WKY and 4 SHR rats)

Control		Kinetics of Ca^2+^ signals compared with the response to the 1^st^ stimulus						
		P2	P3	P4	P5	P6	P7	P8
Half-width	WKY	96.7 ± 1.8	95.2 ± 2.0	95.4 ± 2.4	94.8 ± 2.3	95.5 ± 2.3	92.0 ± 2.0	92.4 ± 2.3
	SHR	94.6 ± 0.8	92.7 ± 0.8	92.9 ± 0.8	95.9 ± 1.3	95.7 ± 1.0	97.4 ± 1.6	96.5 ± 1.6
Normalized Area	WKY	89.0 ± 2.5	84.6 ± 2.6	76.2 ± 2.7	76.2 ± 2.6	70.9 ± 2.2	72.3 ± 3.6	65.1 ± 2.7
	SHR	85.1 ± 1.2	79.0 ± 1.3	78.0 ± 1.6	73.8 ± 1.6	71.9 ± 1.7	67.5 ± 1.6	65.9 ± 1.8

### Occurrence of several Ca^2+^ transient components in rat CCs

Voltage-gated Ca^2+^ transients recorded in rat CCs after a 1-s depolarization revealed details that were previously overlooked: In most CCs, the Ca^2+^ transient begins shortly and comprises a fast component that peaks within approximately 9 s and declines smoothly to baseline in about 10 to 15 s (Fig. 3A and B). Nonetheless, in some CCs, the decay phase last longer (red arrow in Fig. 3B) and displays one or more small bumps (green trace in Fig. 3B), suggesting the persistence of Ca^2+^ fluxes triggered by membrane depolarization. In some instances, the depolarization initiates *two distinct Ca*^*2*+^ *transient components*: an early, fast-rising component that peaks within 2–3 s, followed by a late, slower component that peaks within ~ 10 s. and returns to the baseline in about 11–15 s. Figure 3C and D exemplify recordings obtained from a group of CCs. Some exhibit two Ca^2+^ transient components. The blue triangles signal the early Ca^2+^ transient components. The delay between the early and the late component varies. Sometimes, it is small, making these components difficult to tell apart (see below). The percentage of CCs with two Ca^2+^ transients is relatively small (WKY: *n* = 11/145 (7.6%) and SHR: *n* = 19/150 (12.6%).

### Effects of CPA application on the Ca^2+^ transients of CCs from normotensive WKY rats and hypertensive SHR. Are they affected differently?

**Fig. 5:**
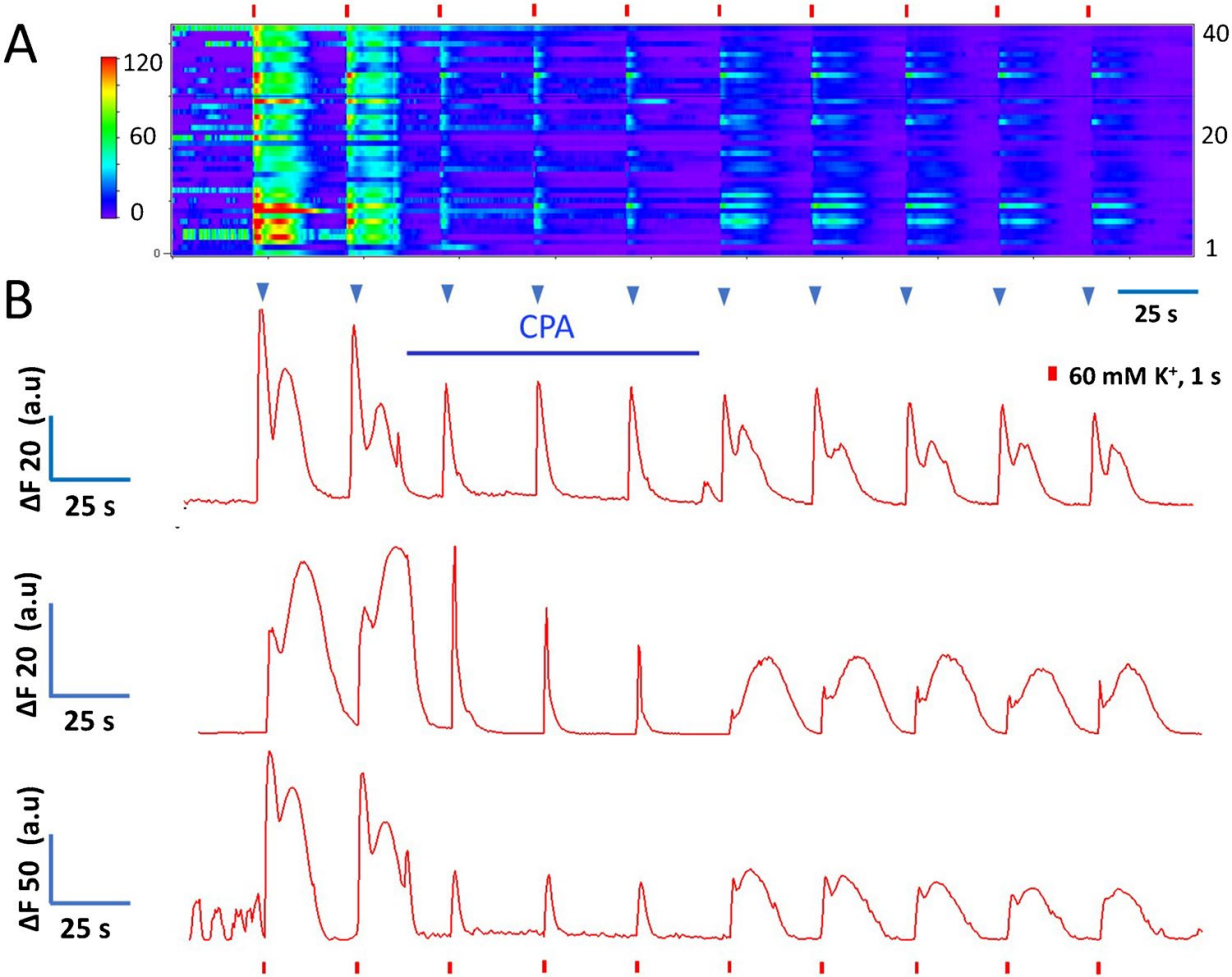
Effects of CPA on voltage-gated Ca^2+^ signals from rat CCs. CCs from the same field were stimulated with eight pulses of high K^+^, 1-s in duration at 30-s intervals, and Ca^2+^ signals produced were recorded before, during, and after applying CPA (10 µM). **A**. Color-coded image representing the Ca^2+^ signals obtained from 43 CCs. Ordinate: cell number, abscissae: time. Fluorescence changes (ΔF = Fi − F0) are displayed in pseudo-color according to the calibration bar on the left. CPA application, indicated by a horizontal line, affects Ca^2+^ signals in all CCs recorded similarly. **B**. Three representative recordings extracted from the data set in **A**, exemplify the attributes of Ca^2+^ transients from individual CCs and the effects of CPA on the kinetics of their Ca^2+^ signals. CPA significantly narrows the Ca^2+^ transient and reduces its amplitude by suppressing the late component. The blue arrowheads signal the early component of each Ca^2+^ signal

Figure 5A is a color-coded image illustrating a group of Ca^2+^ recordings obtained simultaneously from > 40 SHR CCs. One-sec-long depolarizing pulses separated by 30 s were delivered. The first two stimuli were given under control conditions; then CPA was applied, and stimuli 3 to 5 were provided. Then, five more depolarizations were given while the CPA was removed. CPA narrows and reduces the amplitude of Ca^2+^ transient recorded from CCs. However, the extent of CPA inhibition and recovery after CPA varies considerably among cells. In Fig. 5B, the three traces selected from the dataset in Figure 5A exemplify the diverse attributes of Ca^2+^ transients recorded from individual CCs. In particular, these CCs displayed Ca^2+^ transients with early fast and slow delayed components (blue arrowheads indicate the early components). CPA application abolished the late component, affecting less the earlier transient, regardless of the rat strain (SHR or WKY). Similar effects of CPA were observed in five other experiments.

**Fig. 6:**
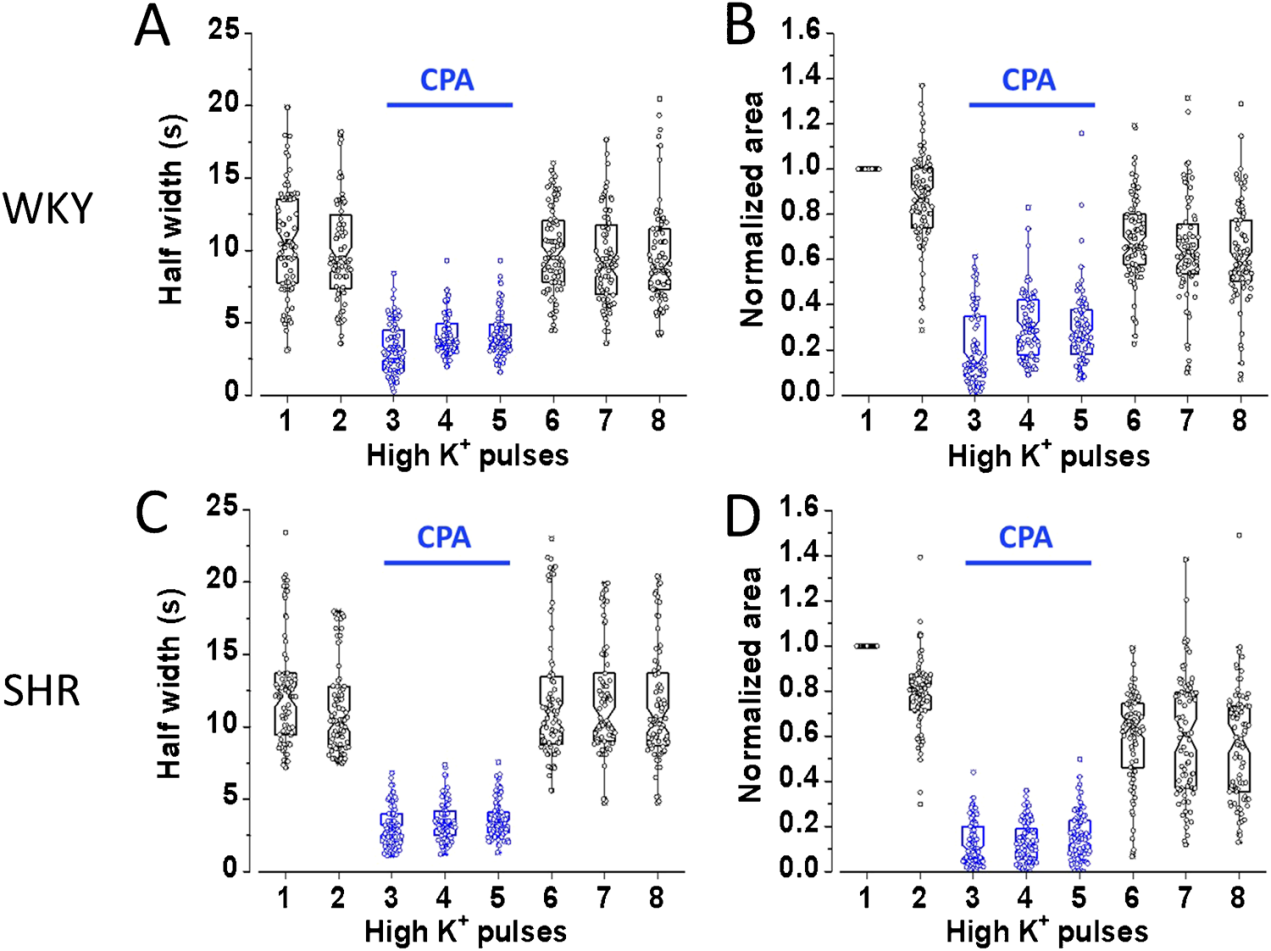
Effects of CPA on a half-width and normalized area of the Ca^2+^ transients from WKY and SHR CCs. CCs were stimulated with eight pulses of high K^+^, 1-s in duration at 30-s intervals. The first two stimuli were given under control conditions; then CPA was applied for 90 s, and stimuli 3 to 5 were provided. Then, CPA was removed, and five more depolarizations were given. **A** and **C**: Hybrid plots of the half-width of the Ca^2+^ transients before, during, and after CPA application in WKY and SHR CCs, respectively. **B** and **D**: Hybrid plots of the normalized area values of the Ca^2+^ transients before, during, and after CPA application in WKY and SHR CCs, respectively (see details in the text and Tables 3 and 4). **A**, **B**: *n* = 80 CCs; **C**, **D**: *n* = 82 CCs

During CPA application, the Ca^2+^ transients narrow drastically (see Fig. 5B). Consequently, the half-width diminished from 10.3 ± 0.3 s (mean of stimuli 1 and 2 ± SEM) to 3.8 ± 0.3 s (mean of stimuli 3 to 5 ± SEM; *n* = 80 CCs, *p* < 0.001) and from 11.9 ± 0.6 s (mean of stimuli 1 and 2 ± SEM) to 3.3 ± 0.2 s (mean of stimuli 3 to 5 ± SEM; *n* = 82 CCs, *p* < 0.001); in WKY and SHR CCs, respectively. These data are summarized in Fig. 6A and C. The percentual reduction of half-width (second *versus* third stimulus) is more significant in SHR CCs (73.1 ± 1.2%) than in WKY CCs (68.5 ± 1.5%; *p* < 0.001, *n* = 80). These effects on half-width reversed almost completely after CPA withdrawal (see Fig. 6A and C; stimuli 6 to 8; and Table 4).

**Table 4:** Inhibition of Ca^2+^ signals by CPA in WKY and SHR CCs. The percentage of half-width (HW) and normalized area (NA) from stimulus-induced Ca^2+^ signals during CPA application (stimuli P3 to P5) is compared to the HW and NA after CPA washout (stimuli 6 to 8), (*n* = 80 WKY CCs and 82 SHR CCs, cultures from 3 WKY and 4 SHR rats)

CPA		Effects of CPA on the kinetics of Ca^2+^ signals compared with the response to the 2^nd^ stimulus					
		CPA			WASH		
		P3	P4	P5	P6	P7	P8
Half-width	WKY	31.5 ± 1.5	41.5 ± 1.6	41.7 ± 1.9	97.8 ± 1.3	93.9 ± 1.3	95.4 ± 1.2
	SHR	26.9 ± 1.2	30.5 ± 1.2	31.9 ± 1.3	94.6 ± 2.2	96.0 ± 1.7	98.7 ± 1.7
Normalized Area	WKY	24.2 ± 2.1	35.5 ± 1.8	33.9 ± 1.9	80.4 ± 2.2	74.6 ± 2.1	71.8 ± 2.1
	SHR	15.5 ± 1.7	16.8 ± 1.8	19.8 ± 1.9	76.1 ± 3.3	77.4 ± 3.0	71.1 ± 2.5

The impact of CPA application on the normalized area of the Ca^2+^ transients is summarized in Fig. 6B and D. The normalized area diminished drastically, from 0.93 ± 0.07 (stimuli 1 and 2; mean ± SEM) to 0.27 ± 0.03 (stimuli 3 to 5: mean ± SEM; *n* = 80 CCs, *p* < 0.01; WKY) and from 0.89 ± 0.10 (stimuli 1 and 2; mean ± SEM) to 0.14 ± 0.01 (stimulus 3 to 5: mean ± SEM; *n* = 80 CCs, *p* < 0.01; WKY and SHR CCs, respectively). Thus, CPA reduces the normalized area by 71% and 84% in WKY and SHR CCs, although the inter-strain difference is not significant (*p* > 0.05). After CPA washout, the half-width shows an almost complete recovery in CCs from both strains (see stimuli 6 to 8 in Fig. 6A and C; see Table 4). In contrast, the normalized area shows a residual CPA effect (see stimuli 6 to 8 in Fig. 6B and D).

Previously we showed that after a membrane depolarization, the Ca^2+^ transients of SHR CCs are ~ 3.1-fold larger in amplitude than WKY CCs due to a more significant contribution of Ca^2+^ release from intracellular stores in these cells [34]. Therefore, we speculated that CPA would affect more the Ca^2+^ dynamics of SHR than WKY CCs. However, we found that CPA similarly inhibited voltage-gated Ca^2+^ transients in CC*s* from *both strains*. CPA had a slightly more potent inhibition of Ca^2+^ signals in SHR CCs than in WKY CCs but not as significant as expected. The inhibitory effects of CPA observed on Ca^2+^ signaling coincide with the effects on catecholamine secretion. Together, these data demonstrate that 1) CPA application affects the exocytotic response and Ca^2+^ transients of rat CCs equally regardless of the strain (SHR or WKY) and 2) Concerning the CPA effects on intracellular Ca^2+^ dynamics, rat CCs behave more similarly to bovine CCs than to mouse CCs. Hereafter, SHR and WKY CCs data are pooled and treated without distinction.

### Effects of CPA application on bovine CCs

**Fig. 7:**
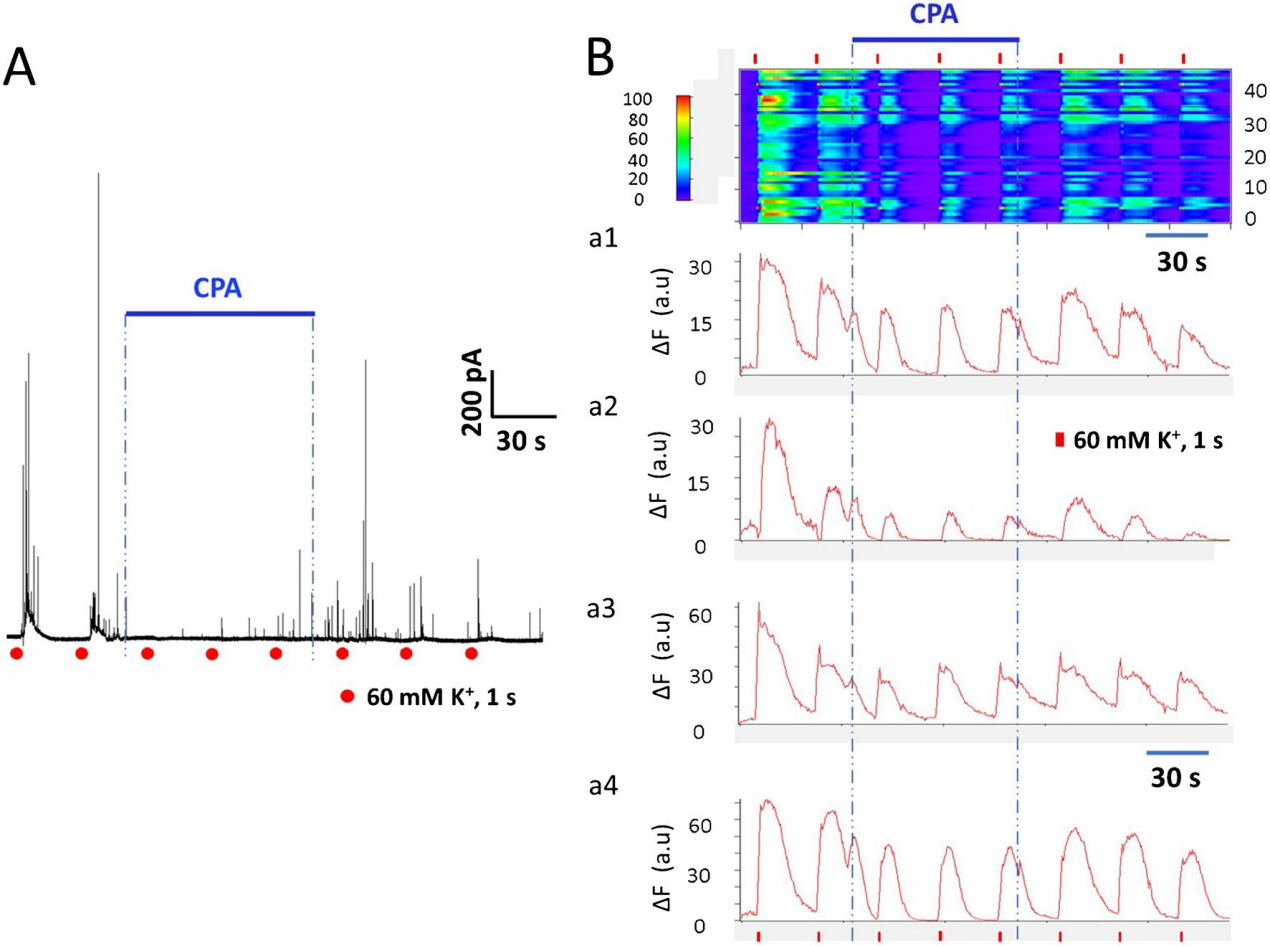
Amperometric and Ca^2+^ signaling recordings from bovine chromaffin cells (BCCs). Effects of CPA. **A**: Example of an amperometric recording from a BCC stimulated using the same protocol as in rat CCs. CPA almost obliterated amperometric responses (see stimulus 3^rd^ through 5^th^); exocytosis rebounded upon CPA washout. **B**: Image representing the Ca^2+^ signals obtained from 45 CCs. Ordinate: cell number, abscissae: time. Fluorescence changes (ΔF = Fi − F0) are displayed in pseudo-color according to the calibration bar on the left. Eight depolarizing stimuli were given, and CPA was perfused from stimuli 3^rd^ through 5^th^. Responses recover partially after CPA washout (stimuli 6^th^ through 8^th^). a1-a4: Four representative traces extracted from the data set in **A **exemplify the attributes of Ca^2+^ transients from individual BCCs, and the effects of CPA on the kinetics of their Ca^2+^ signals. CPA significantly and reversibly reduces the amplitude, half-width, and normalized area of the Ca^2+^ transients (see text)

Pharmacological experiments *In vitro* are susceptible to variations due to perfusion artifacts, different flow rates, local concentration, time in culture, and temperature, so one cannot ascertain that rat CCs behave similarly to bovine CCs without directly comparing species under the same experimental conditions. Thus, we conducted a few experiments in bovine chromaffin cells (BCCs) using the same experimental protocol as rat CCs. Figure 7A exemplifies an amperometric recording from a single BCC stimulated with eight depolarizing pulses. When CPA was perfused, exocytosis was almost entirely obliterated (see stimulus 3^rd^ through 5^th^), and upon CPA washout, the amperometric responses rebounded. Figure 7B exemplifies Ca^2+^ signal recordings obtained from a group of > 40 BCCs. First, two depolarizing stimuli were given under control conditions. When CPA was applied (stimuli 3^rd^ through 5^th^), the amplitude and duration of the Ca^2+^ transients diminished notably, and upon CPA washout, Ca^2+^ transients partially recovered. We did not see many examples of Ca^2+^ transients with early and late components in BCCs. Additional experiments were not performed with BCCs since they are not the main subject of this investigation. Nonetheless, by replicating in our laboratory the effects of CPA on BCCs exocytosis and Ca^2+^ signaling reported by Martínez-Ramírez et al., we confirmed that CPA effects in BCCs resemble those observed on rat CCs, supporting one of the main conclusions of this study.

### Effects of ryanodine application on the Ca^2+^ transients of rat CCs

**Fig. 8:**
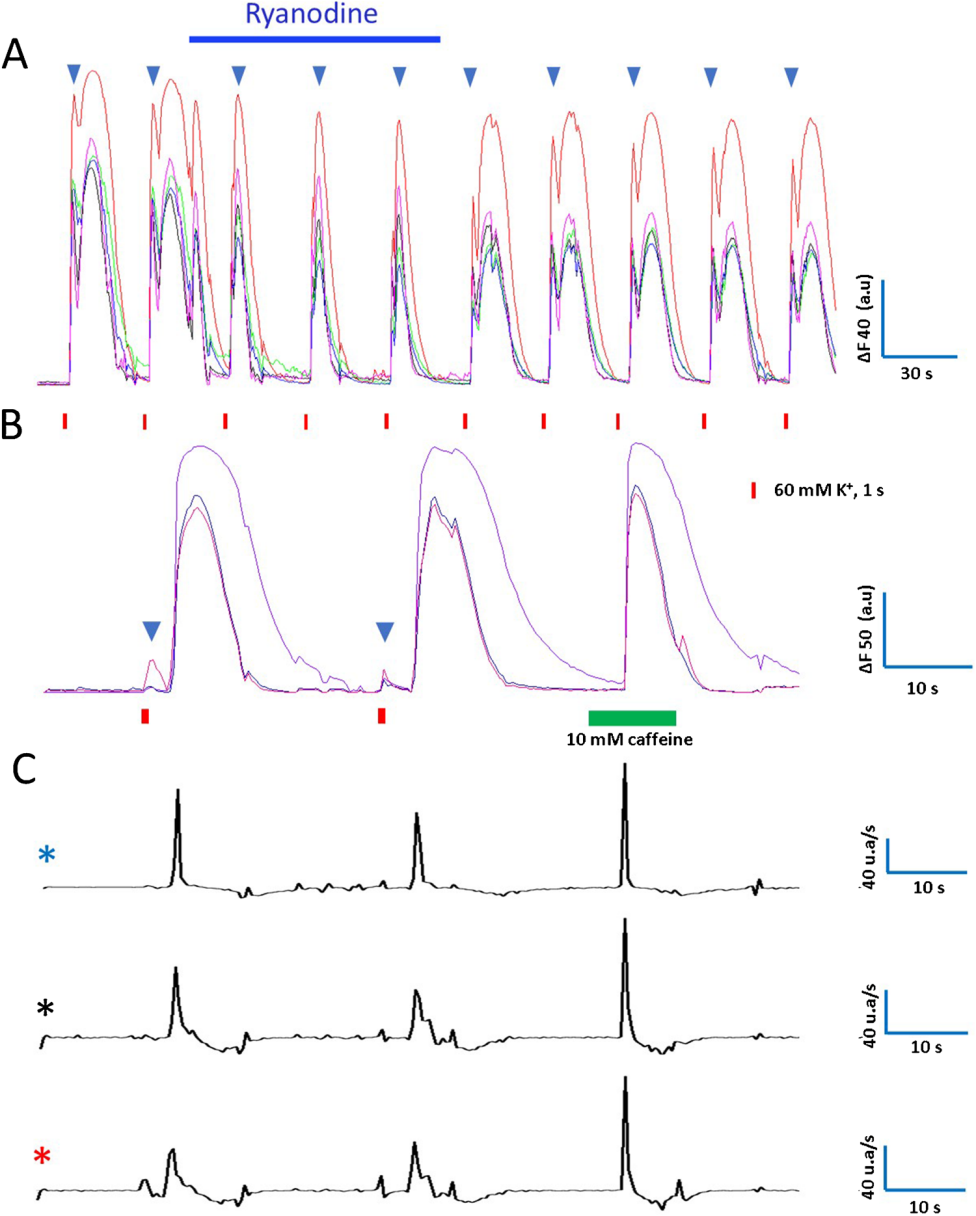
Effects of ryanodine and caffeine on voltage-gated Ca^2+^ signals.** A**. Effects of ryanodine. Five CCs were stimulated repeatedly, and Ca^2+^ signals were recorded before, during, and after applying 10 µM ryanodine for 90 s. These cells display Ca^2+^ transients with early and late components. The blue triangles indicate the early components. Ryanodine narrows the Ca^2+^ transient and abolishes the late component, affecting less the early component. After ryanodine is withdrawn, the late component returns. **B**. Caffeine effects. Ca^2+^ recordings were obtained from three CCs while stimulated with pulses of high K^+^. These cells display early and late Ca^2+^ transient components. Then, caffeine (10 mM) was applied for 10 s, and a major intracellular Ca^2+^ release was produced without an early Ca^2+^ transient. C. The first derivative traces obtained from the Ca^2+^ recordings are shown in **B**. The color of the asterisks indicates the Ca^2+^ trace from which the first derivative was calculated. See the explanation in the text

Figure 8A illustrates an experiment designed to learn more about the mechanisms underlying Ca^2+^ transients of rat CCs. Five CCs were recorded while stimulated with a 1-s-long depolarizing pulse every 30 s. The first stimuli elicit Ca^2+^ transients with two components. The blue triangles signal the early component of each Ca^2+^ transient. Then, ryanodine 10 μM (which specifically blocks ryanodine receptors) was applied for 90 s. This treatment narrows the Ca^2+^ transient and abolishes the late component, affecting less the early component. After ryanodine is withdrawn, the Ca^2+^ transient broadens, and the late component reappears, suggesting its effects are reversible after short-term exposure. The predominant inhibition of the late Ca^2+^ transient by ryanodine and CPA, even though these two agents act by different mechanisms, supports the notion that the delayed component mainly represents the contribution of CICR (see Discussion).

**Fig. 9:**
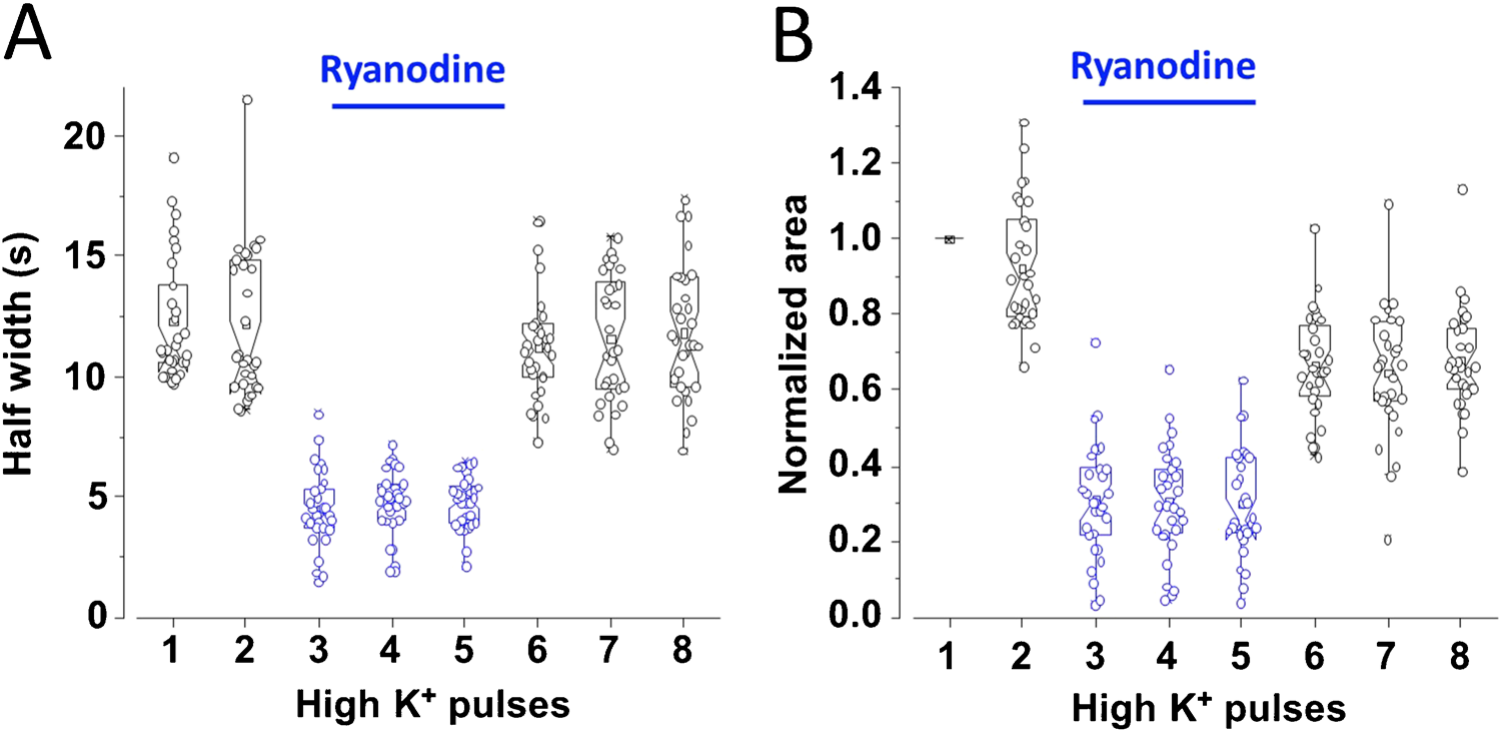
Effects of ryanodine on the half width and normalized area of voltage-gated Ca^2+^ signals. Rat CCs were stimulated with eight pulses of high K^+^, 1-s duration at 30-s intervals. The first two stimuli were given under control conditions; then ryanodine 10 µm was applied for 90 s, while stimuli 3 to 5 were supplied. Then, ryanodine was discontinued, and five more depolarizations were given. **A** and **B**: Hybrid plots of the half-width (**A**) and normalized area (**B**) of the Ca^2+^ transients before, during, and after ryanodine application. (see details in the text and Tables 3 and 4). **A**: *n* = 30 CCs, **B**: *n* = 30 CCs

Selective blockade of the voltage-gated Ca^2+^ influx to demonstrate its participation in the early and delayed Ca^2+^ transients is pointless because it completely suppresses the intracellular Ca^2+^ signal (data not shown). Nonetheless, caffeine can trigger intracellular Ca^2+^ release by itself. Figure 8B shows Ca^2+^ recordings from three CCs. The first two stimuli are depolarizing pulses which produce a small, early Ca^2+^ transient (blue triangles) followed by a prominent, late component. Then, caffeine (10 mM, 10 s) was applied, and after a delay of approximately 5-s, an intracellular Ca^2+^ release was produced. The kinetics of the caffeine-induced Ca^2+^ signal resembles that of the late component but with a faster rate of rise and decline (Fig. 8C) and without an early Ca^2+^ transient component. The long delay of about 5-s is characteristic of the caffeine responses, as reported earlier [34]. Additional experiments were conducted to analyze ryanodine effects further. We used the same protocol as Figs. 5 and 6 but with ryanodine instead of CPA. As summarized in Fig. 9, ryanodine reversibly reduced the half-width and the normalized area of the Ca^2+^ transients similarly to CPA. The narrowing of the Ca^2+^ transients by CPA and ryanodine suggests that the late component is mainly due to CICR**.**

During ryanodine application, the Ca^2+^ transients narrow drastically (see Fig. 8A). Consequently, half-width diminished from 12.3 ± 0.1 s (mean of stimuli 1 and 2 ± SEM) to 4.7 ± 0.1 s (mean of stimuli 3 to 5 ± SEM; *n* = 30 CCs, *p* < 0.01). The percentual reduction of half-width (second *versus* third stimulus) is 63 ± 1.9% (*p* < 0.01, *n* = 30). These data are summarized in Fig. 9A. Ryanodine effects on half-width reversed after its withdrawal (see Fig. 9A stimuli 6 to 10). The impact of ryanodine application on the normalized area of the Ca^2+^ transients is summarized in Fig. 9B. Normalized area diminished drastically, from 96 ± 3.6% (stimuli 1 and 2; mean ± SEM) to 30.6 ± 0.3% (stimuli 3 to 5: mean ± SEM; *n* = 30 CCs, *p* < 0.01). The percentual reduction of normalized area (second *versus* third stimulus) is 66.4 ± 2.9%. After ryanodine washout, the normalized area recovers partially (see stimuli 6 to 8 in Fig. 9B). Similarly, the normalized area shows a residual effect after CPA (see stimuli 6 to 8 in Fig. 6B, D).

### Simultaneous recording of Ca^2+^ signaling and amperometric spikes from single CCs

**Fig. 10:**
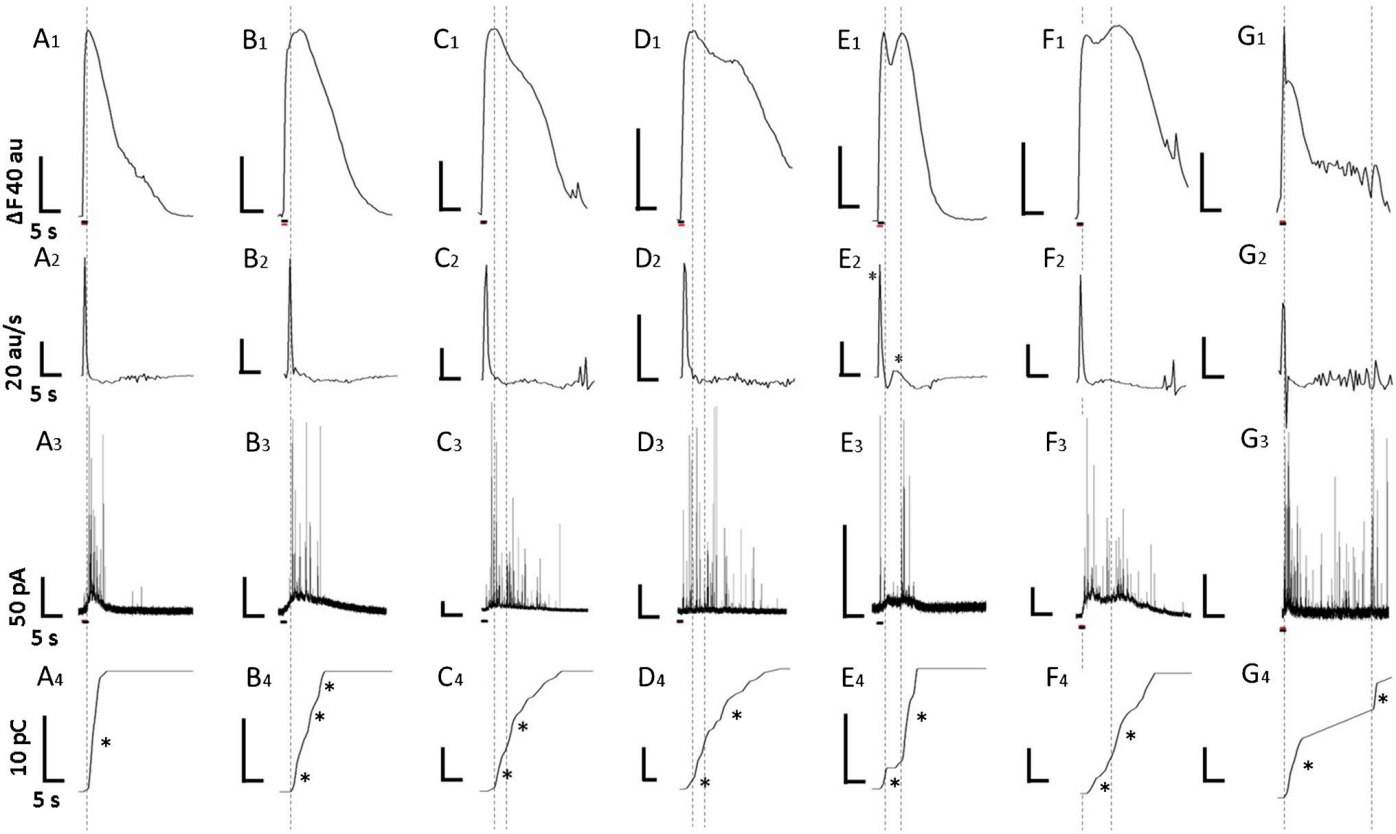
Simultaneous recording of Ca^2+^ signaling and amperometric spikes from single CCs. The Ca^2+^ signal in response to the first depolarizing stimulus of seven representative CCs was collected (A1-G1), and correlated with the corresponding first derivative of the Ca^2+^ signal (A2-G2), the rate of generation of amperometric spikes (A3-G3), and the building-up of cumulative charge over time (A4-G4). Three patterns of Ca^2+^ signaling were recognized in rat CCs: Type 1: A narrow Ca^2+^ transient with a fast-rising phase and smooth decay phase (A1). Type 2: A Ca^2+^ transient of longer duration with occasional humps or bumps in the decay phase (B1, C1, D1), and Type 3: A Ca^2+^ signal with two distinct Ca^2+^ transients (E1, F1). An atypical CC (G1) with an early fast Ca^2+^ transient, a hump, and a plateau with increasing oscillations is also included. Explanation in the text

In some rat CCs, a brief membrane depolarization triggers two discharges of amperometric spikes (see Figs. 1A, B and 2A, B; black asterisks). Accordingly, some CCs exhibited Ca^2+^ transient with early and late components. We wondered if these two phenomena could be related. Simultaneous amperometric and Ca^2+^ signaling recordings were performed to correlate the kinetics of the Ca^2+^ transient with the rate of generation of amperometric spikes. Since bursts of amperometric spikes can be difficult to discern, we computed the building-up of cumulative charge over time. We also calculated the first derivative of the Ca^2+^ signal to evaluate the sign and intensity of the Ca^2+^ fluxes involved. Figure 10 illustrates the result of the analysis of the response to a brief depolarizing stimulus of seven representative CCs. Three patterns of Ca^2+^ signals were identified:

- Type 1: A narrow Ca^2+^ transient with fast-rising and smooth decay (A1), which gave a single positive peak in the first derivative of the Ca^2+^ signal (A2), a compact single burst of amperometric spikes (A3), and a single stepwise increase in cumulative charge (A4).
- Type 2: A broadened Ca^2+^ transient with occasional bumps in the decay phase. Figures B1, C1, and D1 exemplify recordings from three CCs with progressively wider Ca^2+^ signals. This type of Ca^2+^ signaling correlates with a single positive peak in the derivative of the Ca^2+^ signal (B2, C2, and D2), a robust initial burst of amperometric spikes followed by a low-frequency spike firing, sometimes with additional short bursts (B3, C3, and D3), and a stepwise increase in cumulative charge followed by one or more smaller increases (B4, C4, and D4).
- Type 3: A Ca^2+^ transient with two components. Figures E1 and F1 exemplify this type of Ca^2+^ signaling which correlates with two positive peaks in the first derivative of the Ca^2+^ signal (E2 asterisks, F2), two discernable surges of amperometric spikes (E3, F3;) and two stepwise increases in cumulative charge (E4, F4; asterisks).

One of the CCs from this set showed an atypical pattern: An early fast Ca^2+^ transient, followed by a hump and a plateau, with oscillations of increasing size at the end (G1). The derivative of this Ca^2+^ signal consists of a prominent peak and several small fluctuations (G2). The amperometric recording shows a robust burst of spikes followed by a continuous emission of spikes and another short spurt near the end, correlated with the Ca^2+^ oscillations. The cumulative charge shows a stepwise increase, a ramp-like during the continuous spike firing, and a smaller stepwise increase near the end. This analysis confirms that the kinetic characteristics of the intracellular Ca^2+^ signal determine the attributes of the amperometric response.

### Simultaneous recording of Ca^2+^ signaling and amperometric spikes from single CCs before during, and after CPA application

**Fig. 11:**
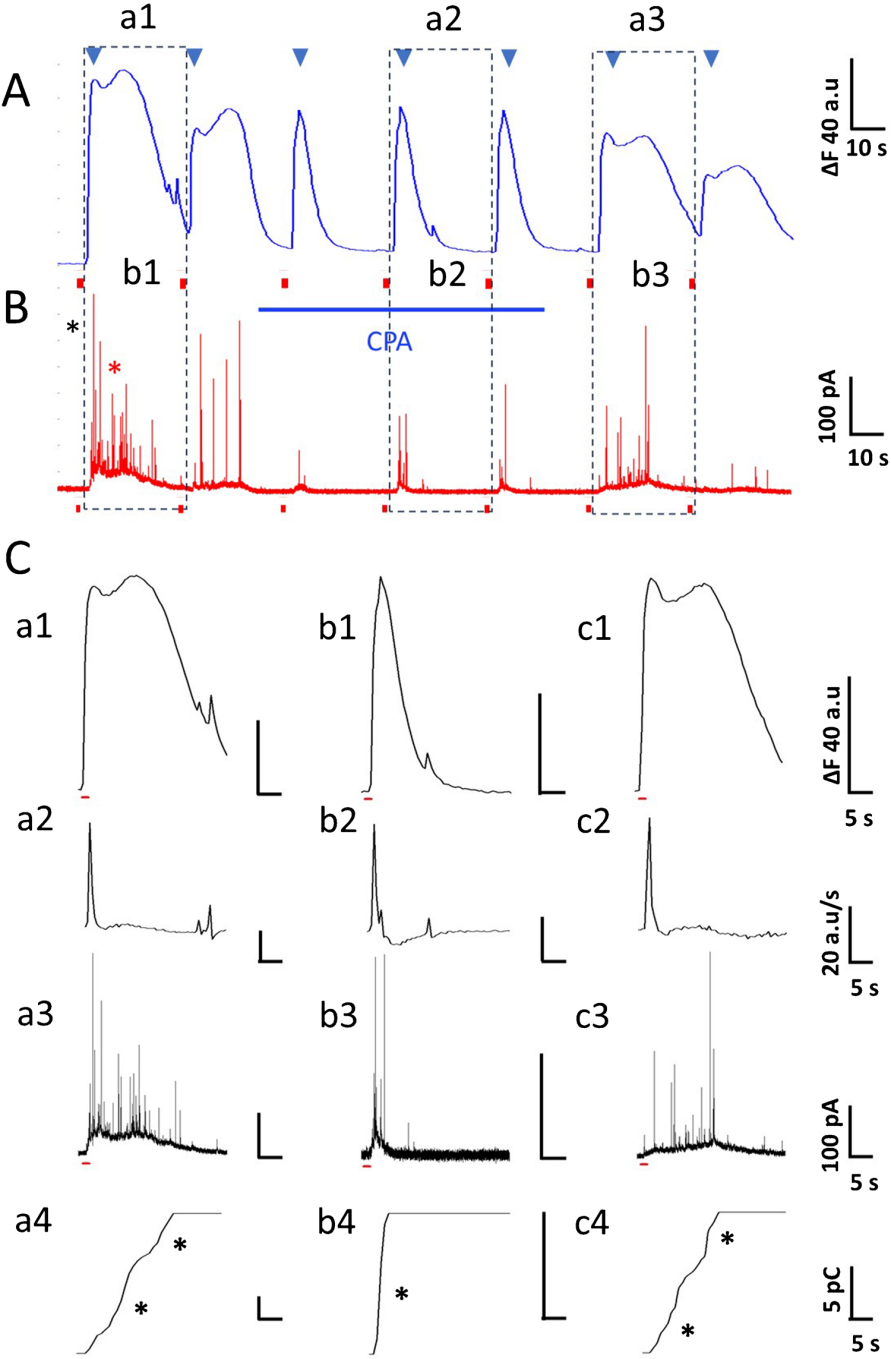
Amperometric and Ca^2+^ signaling recording from the same CCs before, during, and after the CPA application. **A**, **B**: Ca^2+^ transients and amperometric spikes were elicited by repeated stimulation. The first depolarizing stimuli elicit double Ca^2+^ transients nearly fused (11Aa1). During CPA application, the Ca^2+^ transient narrows and the late component dissapears (11Aa2). After CPA, the double Ca^2+^ transients reappear (11Aa3). The corresponding amperometric recording shows a double burst of spikes (Fig. 11Bb1; black and red asterisks). The narrowed Ca^2+^ transients elicit a small compact burst of amperometric spikes (11Bb2). After CPA, the amperometric recording again shows two bursts of spikes (11Bb3). **C**: Analysis before during and after CPA. The control double peak Ca^2+^ transient (11Ca1) correlates with a large peak and a hump in the first derivative (11Ca2), two bursts of amperometric spikes (11Ca3), and a cumulative charge with two stepwise increases (11Ca4). During CPA, the narrow Ca^2+^ transient (11Cb1) correlates with a single spike in the first derivative (11Cb2), a single compact amperometric discharge (11Cb3), and a stepwise increase in cumulative charge (11Cb4). After CPA, the Ca^2+^ transient shows a double peak (11Cc1), a large spike and a hump in the first derivative (11Cc2), two bursts of amperometric spikes (11Cc3), and two stepwise increases in cumulative charge (11Cc4). Explanation in the text

As proof of concept, amperometric spikes and Ca^2+^ signaling were recorded before, during, and after the CPA application from a CC exhibiting two Ca^2+^ transients. The first depolarizing stimulus elicited a large Ca^2+^ transient with two components nearly fused (Fig. 11Aa1), and the amperometric recording shows two bursts of spikes (Fig. 11Bb1; black and red asterisk). The second stimulus, 30 s later, elicited a smaller double Ca^2+^ transient that only produced continuous spike firing at low frequency. During CPA application, the late component is abolished, and the remaining early Ca^2+^ transient (Fig. 11Aa2) elicits a smaller amperometric response (Fig. 11Bb2). The late Ca^2+^ transient component reappears when CPA is withdrawn (Fig. 11Aa3), and the amperometric recording shows two bursts of spikes (Fig. 11B). The last Ca^2+^ transient significantly diminishes amplitude and triggers almost no spikes.

We applied to these recordings the same analysis as in Fig. 10. Here, the Ca^2+^ transient amplitudes are normalized for comparison. As shown in Fig. 11C, the first Ca^2+^ transient, with early and late components (Fig. 11Ca1), gives a first derivative with a large peak followed by a hump (Fig. 11Ca2); two bursts of spikes in the amperometric recording (Fig. 11Ca3) and two stepwise increases in the cumulative charge (Fig. 11Ca4; asterisks). During the CPA application, the late component is abolished, resulting in a smaller, narrower Ca^2+^ transient (Fig. 11Cb1), a single peak in the first derivative trace (Fig. 11Cb2), a single compact amperometric discharge (Fig. 11Cb3) and a stepwise increase in the cumulative charge (Fig. 11Cb4, asterisk). After CPA is withdrawn, the Ca^2+^ transient shows again early and late components (Fig. 11Cc1), the first derivative had a single spike followed by a tiny hump (Fig. 11Cc2), and the amperometric response shows two bursts of spikes. (Fig. 11Cc3). (Fig. 11Ca3), and the cumulative charge shows two stepwise increases (Fig. 11Cc4) again.

This new analysis not only supports the main conclusion of this part of the study but also provides a new dimension to the dynamics of exocytosis in chromaffin cells: the existence of a robust and predictable correlation between the kinetics of the whole-cell intracellular Ca^2+^ signal and the rate of exocytosis at the single cell level.

## Discussion

Substantial differences have been reported in Ca^2+^ dynamics and exocytosis in CCs from several mammalian species [15]. Bovine chromaffin cells express an efficient mechanism of Ca^2+^-induced Ca^2+^ release (CICR) [3]. Rigual et al. 2002 reported that mouse CCs lack ryanodine receptor-mediated Ca^2+^-induced Ca^2+^ release (CICR) [30]. However, recent studies demonstrated the presence of ryanodine receptors and CICR in CCs from C57BL/6 mice [38]. In a recent publication, Martínez-Ramírez et al. reported the effects of acute SERCA pump inhibition with CPA on the exocytotic responses and intracellular Ca^2+^ signals elicited by ACh in mouse and bovine CCs [25]. In mouse CCs, CPA *enhanced* exocytosis, but in bovine CCs, it i*nhibited* exocytosis. After CPA washout, exocytosis diminished in mouse CCs, while it bounced back in bovine CCs. To explain these discrepancies, the authors suggested that differences in [Ca^2+^]i handling by the endoplasmic reticulum and mitochondria could affect vesicle traffic and refill of the secretory granule's rapid release pool. This assumption is based on the fact that mitochondria capture more Ca^2+^ in mouse CCs than in bovine CCs [2]. However, mitochondrial Ca^2+^ transport alone cannot account for these differences because CPA does not affect these organelles [7, 14]. The discrepancies can be better explained by assuming that mouse and bovine CCs differ in the relative strength of opposing ER functions: Ca^2+^ uptake and Ca^2+^ release: mouse CCs display weak Ca^2+^ release and strong Ca^2+^ sinking [30]. The reverse is true for bovine CCs [3]. CPA inhibits both mechanisms: Ca^2+^ uptake directly and CICR indirectly [18, 25].

This work addresses some critical questions raised by the study of Martínez-Ramírez et al. One important aspect is that stimulation of exocytosis of CCs with ACh may be hard to interpret because of the combined action of nAChRs, mAChRs, and voltage-gated Ca^2+^ channels [38]. We performed similar experiments in rat CCs using high-K^+^ depolarization, which only involves voltage-gated Ca^2+^ channels. Also, given the crucial role of CICR in this context, we compared the effect of CPA (which blocks the SERCA pump) and ryanodine (which blocks RyRs). Intriguingly, Martínez-Ramírez et al. reported that the amplitude of ACh-induced Ca^2+^ signals diminished only slightly with CPA in mouse and bovine CCs [25]. The reasons for the conflicting results between amperometry and Ca^2+^ signals need to be addressed. We also wonder if CPA affects rat's CCs differently depending on if they belong to normotensive WKY or hypertensive SHR.

### Dynamics of CA exocytosis after repeated stimulation normotensive WKY and hypertensive SHR CCs under control conditions

As reported earlier, SHR CCs produce more exocytotic events per stimulus than WKY CCs (compare Fig. 1A, B), and the kinetic parameters of the amperometric spikes recorded from both types of CCs are also different (see Table 1). These differences in the rate of CA secretion between SHR and age-matched WKY rats are already present since the prehypertensive stage [28]. The generation of larger Ca^2+^ signals due to a more robust amplification by Ca^2+^-induced Ca^2+^ release can explain the increased CA secretion in SHR CCs [34]. A relevant aspect is that these measurements and comparisons have been carried out by looking at the response to a single stimulus. Accordingly, the number of spikes and the cumulative charge elicited by the first stimulus is two-fold and three-fold greater in SHR compared to WKY CCs (see Figs. 1C and D). We recently reported that SHR CCs have 82% more granules per cytoplasmic area than WKY CCs [28], which suggests that, besides the larger Ca^2+^ signals, the enhanced secretion of catecholamine in SHR CCs could result from a higher density of secretory granules in the cytoplasm, making exocytosis more likely.

Nonetheless, the number of spikes and the cumulative charge fall considerably (35% to 65%) between the first and second stimuli and even more with subsequent stimuli. Therefore, after the fifth stimulus, the inter-strain difference in peak cumulative charge and number of spikes per stimulus *becomes negligible* (see Figs. 1C, D, and Table 1). The finding that CA hypersecretion reported in SHR CCs becomes less marked or disappears after recurrent stimulation might be relevant to the possible role of CCs in the physiopathology of hypertension. Both parameters decay after the first stimulus in mouse and bovine CCs stimulated at 60-s intervals, with less decay in bovine than in mouse CCs [25].

Why do CCs secrete fewer catecholamine granules after the first stimulus? It is well established that the magnitude and duration of the intracellular Ca^2+^ signals regulate the distribution of chromaffin granules among the various pools and their mobilization, docking, priming, and fusion with the plasma membrane [16, 24]. However, a reduction in the size of the Ca^2+^ transient can be ruled out because Ca^2+^ transients in SHR and WKY CCs do not decline abruptly with repeated stimulation (see Figs. 3A and C). The immediate and ready-releasable pools are a small group of chromaffin granules located near the voltage-gated Ca^2+^ channels at the plasma membrane [5]. Perhaps unstimulated SHR CCs contain more granules in the immediate and ready-releasable than WKY CCs [36]. The first stimulus depletes these pools, causing subsequent stimuli to be significantly less effective [35]. The depletion of supplementary granules from the immediate and ready-releasable pools with the first stimulus could also explain why the inter-strain difference in the number of spikes and the peak cumulative charge disappears after repeated stimulation (see Figs. 1C and D, Table 1). Both parameters decay after the first stimulus in mouse and bovine CCs stimulated at 60-s intervals, with less decline in bovine than in mouse CCs [25].

### Effects of CPA on the catecholamine exocytosis of CCs from SHR and WKY rats

We reported earlier that the depolarization-induced CA secretion is more significant in SHR CCs than in WKY CCs due to a more substantial contribution of the Ca^2+^-induced Ca^2+^ release [34]. Based on these results, we assumed that in SHR CCs, where Ca^2+^ release from intracellular stores is robust, CPA should i*nhibit* the amperometric responses (like in bovine CCs). In contrast, in WKY CCs, where Ca^2+^ release from intracellular stores is weak, CPA should affect less the amperometric responses (like in mouse CCs). CPA drastically reduces the amperometric responses *in both rat strains* (Figs. 2A and B). The effects began rapidly and persisted while CPA was present. After CPA was withdrawn, the amperometric responses recovered. This result is unexpected because CPA application *increased slightly* ACh-induced exocytosis in mouse CCs, a closely related species [25]. After CPA treatment, more amperometric spikes were elicited than in control CCs (compare responses to stimulus 6–8 in Figs. 1A, B and 2A, B), probably because during CPA application, the less effective stimuli caused less depletion of the ready-releasable pool and the Ca^2+^ content of the ER which would replenish promptly after SERCA pump recovers. This response resembles the "rebound" response observed in bovine CCs after CPA removal [25]. The magnitude of this rebound is similar in WKY and SHR CCs. Together, these results demonstrate that 1) CPA application strongly inhibits the exocytotic response of CCs regardless of the rat strain (SHR and WKY) and 2) SERCA pump inhibition by CPA affects CA secretion of rat CCs more similarly to bovine *CCs* than mouse CCs, despite the vast phylogenetic distance to the most recent common ancestor between rat and cow (94 million years ago), compared with a smaller distance between mouse and rat (10.3 million years ago; [20]).

### Depolarization-induced Ca^2+^-rises in CCs from normotensive WKY rats and hypertensive SHR under control conditions

An intriguing result from the study by Martínez-Ramírez is that in bovine CCs, ACh-induced exocytosis is inhibited significantly during CPA application, *but the Ca*^*2*+^ *transients recorded under the same conditions diminished only slightly*, both in mice and bovine [25]. The discrepancy of CPA effects between amperometry and Ca^2+^ signaling could be due to the [Ca^2+^]i sensitive dye used. Fura-2 is a high-affinity (220 nM) [Ca^2+^]i selective fluorescent indicator that saturates at low micromolar concentrations and interferes with intracellular Ca^2+^ dynamics (i.e., antagonizes CICR; [4]). Instead, we performed imaging experiments with the [Ca^2+^]i indicator Fluo-4 because it has a lower Ca^2+^ affinity (~ 350 nM), can measure greater Ca^2+^ elevations without saturation, and does not interfere with Ca^2+^ dynamics. As demonstrated in Figs. 3A and B, the peak amplitude of the Ca^2+^ transients in response to 1-s-long depolarizing stimuli, separated by 30-s intervals, declines slightly and steadily. Conversely, the amperometric responses decrease step-like after the first stimulus and diminish gradually after that (cf. Fig. 1C and D).

The slight use-dependent decline in Ca^2+^ mobilization observed here is likely due to the accumulation of voltage-dependent inactivation of Ca^2+^ channels since there is no perceptible build-up of [Ca^2+^]i. The question remaining is why, if the Ca^2+^ transient elicited the second pulse is almost identical to the first one, the amperometric response is severely reduced. One possibility is that unstimulated SHR CCs contain more granules in the immediate and ready-releasable pools than WKY CCs [36]. A significant granule depletion caused by the first stimulus explains the smaller amperometric responses from subsequent stimuli [35]. This interpretation can be tested directly in future experiments.

Ca^2+^ transients elicited by 1-s stimulation showed kinetic details previously overlooked: In most CCs, the Ca^2+^ signal comprises a single Ca^2+^ transient component that decays promptly (Figs. 3A, B). In some cells, the decay phase last longer or displays one or more bumps, suggesting long-lasting Ca^2+^ fluxes triggered by the stimulus. The stimulus may also trigger an early, fast-rising component followed by a late, slower component (Figs. 3C, D).

### Effects of CPA and ryanodine application on the Ca^2+^ transients from rat CCs from normotensive WKY and hypertensive SHR rats

In CCs where the Ca^2+^ transients only had one Ca^2+^ transient, CPA application significantly reduced both the half-width and the normalized area of the Ca^2+^ transients. In CCs with widened Ca^2+^ transient or two Ca^2+^ transients, CPA shortened the duration and abolished the late component, if present. After CPA was withdrawn, the Ca^2+^ transient broadened, and the late part recurred, somewhat diminished. The interpretation of these results is straightforward: SERCA pump inhibition by CPA leads to Ca^2+^ depletion of intracellular stores, reducing the gain of the CICR mechanism. Suppression of the late Ca^2+^ transient component, if present, suggests that it relates to Ca^2+^ signal amplification by CICR. The early event, mainly corresponding to the voltage-gated Ca^2+^ influx, is less inhibited since CPA does not directly affect Ca^2+^ channels. Nonetheless, a small contribution of CICR to this early Ca^2+^ transient cannot be excluded.

During repeated stimulation of rat CCs, the half-width and the normalized area of the Ca^2+^ transient remain relatively stable regardless of the strain (Fig. 4A-D). On the other hand, during CPA application, the Ca^2+−^ transients diminished in amplitude while the half-width and the normalized area fell drastically (Figs. 6A-D). These effects of CPA are reversed partially after its withdrawal. Concerning a possible differential impact of CPA between WKY and SHR CCs, our results show that CPA effectively inhibited the half-width and the normalized area of voltage-gated Ca^2+^ transients in CCs *regardless of the strain*. These data agree with the observation that CPAs strongly inhibited catecholamine exocytosis in CCs from both subspecies*.* As anticipated, the reduction in the half-width and the normalized area of the Ca^2+^ transients is proportionately more significant in SHR CCs than in WKY CCs, suggesting a stronger CICR in SHR CCs, as previously demonstrated [34]. Together, these results indicate that CPA application similarly affects the exocytotic response and voltage-gated Ca^2+^ transients of rat CCs (SHR or WKY). By replicating in our laboratory the effects of CPA on voltage-gated exocytosis and Ca^2+^ dynamics from bovine CCs (Fig. 7), we confirmed the earlier results by Martínez-Ramírez et al. [25] supporting one of the main conclusions of this study:  that the effects of CPA on exocytosis and intracellular Ca^2+^ dynamics in rat CCs behave more similarly to bovine CCs than mouse CCs, despite the vast phylogenetic distance from the most recent common ancestors.

Incubation with the RyR blocker ryanodine (10 μM) diminished the depolarization-induced CA secretion by 77% in SHR CCs and only 10% in WKY CCs [34], demonstrating that RyRs-mediated intracellular Ca^2+^ release has a more significant contribution in SHR CCs to CA exocytosis [34]. Nonetheless, the impact of ryanodine on intracellular Ca^2+^ signaling in rat CCs from these two strains had not been examined in detail. As shown in Fig. 8A, a brief exposure to ryanodine 10 μM abolished the late Ca^2+^ transient component, nearly sparing the early component. Similar results were obtained after incubating the cells for 10 min with the fast intracellular Ca^2+^ chelator BAPTA-AM (10 μM), which also interferes with Ca^2+^-induced Ca^2+^ release (data not shown; see [19]). The reciprocal experiment (the application of caffeine, which *opens* RyRs) triggers a robust Ca^2+^ release from intracellular stores that resembles the late Ca^2+^ transient. Nonetheless, the caffeine response occurs with a characteristic delay, and the kinetics of rise and fall are faster than the responses to membrane depolarization (compare first derivative traces in Fig. 8C). The predominant inhibition of the late Ca^2+^ transient by ryanodine and CPA (two agents with different mechanisms of action) supports the notion that the delayed component mainly corresponds to the contribution of CICR and that the early and late Ca^2+^ signal components represent independent Ca^2+^-signaling phenomena.

How do the effects of ryanodine and CPA compare? The impact of ryanodine on the half-width and normalized area of the Ca^2+^ transients of rat CCs is summarized in Fig. 9B, and the effects of CPA are summarized in Figs. 6A, B, and C, D. In general, the actions of ryanodine and CPA resemble. However, CPA inhibition is somewhat more potent: it reduces the half-width by 73.1% and 68.5% and the normalized area by 84% and 71% in SHR and WKY CCs, respectively. In contrast, ryanodine's peak reduction of the half-width is 63%, and of the normalized area, 66%. After CPA washout, the half-width almost completely recovers in CCs from both strains (Figs. 6A and C), while the normalized area shows a residual CPA effect (Figs. 6B and D). Similarly, ryanodine effects on the half-width reversed after its withdrawal (see Fig. 9A), but the normalized area recovers only partially (see Fig. 9B). These differences might be related to the observation that the half-width of the Ca^2+^ transients remains relatively stable during recurrent stimulation (Figs. 4A and C). At the same time, the normalized area declines gradually, reaching 23.9% (WKY) and 19.2% (SHR) decay after eight stimuli (Figs. 4B and D).

### Simultaneous recording of Ca^2+^ signaling and amperometric spikes from single rat CCs

In some amperometric recordings, we noticed that in response to a brief depolarization, the initial burst of amperometric spikes was followed by one or more discharges of spikes (see Figs. 1A, B and 2A, B; black asterisks). We wondered if this exocytosis pattern could be associated with the early and late components of the Ca^2+^ signal observed in some CCs. The presence of several bursts of spikes in the amperometric recordings is difficult to ascertain by eye. Nonetheless, by computing the integral over time of the amperometric recording, it is possible to correlate bursts of exocytosis with stepwise increases in cumulative charge. We conducted simultaneous amperometric and Ca^2+^ signaling recordings to correlate the kinetics of the Ca^2+^ transient, the rate of generation of amperometric spikes, and the building-up of the cumulative charge. For this analysis, we also computed the first derivative of the Ca^2+^ signal to evaluate the magnitude of the Ca^2+^ fluxes involved. Figure 10 summarizes the assessment of the responses of seven representative CCs to a single depolarizing stimulus.

Three patterns of Ca^2+^ signaling were recognized in rat CCs: Type 1: A narrow Ca^2+^ transient with a fast-rising phase smooth decay phase. Type 2: A Ca^2+^ transient of longer duration with occasional humps or bumps in the decay phase. Type 3: A Ca^2+^ signal with two distinct Ca^2+^ components. In all the cell types, the first derivative of the Ca^2+^ signal showed a positive peak during the rising phase and a smaller negative deflection during the decay phase. In Type 2 cells, small additional fluctuations could be seen near the end, and in Type 3 cells, two distinct positive peaks could be readily recognized. All CCs showed an early compact single burst of amperometric spikes. Type 2 CCs, besides the early burst of amperometric spikes, displays a low-frequency spiking and additional short bursts. Type 3 CCs show early and late surges of amperometric spikes. Regarding the building-up of the cumulative charge, in all the cell types, the early compact burst of spikes caused a significant single-step increase in cumulative charge. In Type 2 cells, the cumulative charge shows one or more additional smaller stepwise rises near the end, and in Type 3 cells, two distinct stepwise increases in incremental charge can be readily recognized.

The analysis of a CC with an atypical signaling pattern is also shown in Fig. 10: The early sharp Ca^2+^ rise is followed by a hump, a sustained plateau, and oscillations of increasing size near the end (Fig. 10G1). The derivative shows an early spike and several small fluctuations near the end. The amperometric recording displays an early burst of spikes, a continuous low-frequency eruption, and another short burst near the end. The cumulative charge trace shows a stepwise increase at the beginning, a ramp-like increase during the continuous spike firing, and a smaller stepwise increase at the end. These results support the notion that the kinetic characteristics of the intracellular Ca^2+^ signal predictably determine the attributes of the amperometric response.

### Simultaneous recording of Ca^2+^ signaling and amperometric spikes from single CCs before, during, and after CPA application

As proof of concept of the ideas presented here, amperometric spikes and Ca^2+^ signaling were simultaneously recorded in rat CCs before, during, and after the CPA application (Figs. 11A and B). The first depolarizing stimulus elicits a large Ca^2+^ transient, with early and late components nearly fused (Fig. 11Aa1). The corresponding amperometric recording below (Fig. 11Bb1) shows a double burst of spikes (black and red asterisks). The second stimulus, 30 s later, also elicited a double Ca^2+^ transient but smaller, and only a train of low-frequency spikes was produced. During CPA application, the late component of the Ca^2+^ transients is abolished (Fig. 11Aa2), affecting less the early component. These narrowed Ca^2+^ transients only elicit a few amperometric spikes (see Fig. 11Bb2). After CPA withdrawal, the late Ca^2+^ signaling component reappears (Fig. 11Aa3), and the amperometric recording again shows two bursts of spikes (Fig. 11Bb3. The last stimulus, which elicited a weak Ca^2+^ transient, triggered very few amperometric spikes.

 In Fig. 11C, the control Ca^2+^ transient (Fig. 11Ca1) with early and late elements correlates with a first derivative with a single spike followed by a small hump (Fig. 11Ca2), an amperometric recording with two bursts of spikes (Fig. 11Ca3), and a cumulative charge with two stepwise increases (Fig. 11Ca4). CPA application abolished the late wave, leaving a smaller, narrower Ca^2+^ transient (Fig. 11Cb1), a first derivative with a single spike (Fig. 11Cb2), a brief amperometric discharge (Fig. 11Cb3), and a cumulative charge showing a single stepwise increase (Fig. 11Cb4). After CPA is withdrawn, the Ca^2+^ transient shows again early and late components (Fig. 11Cc1), the first derivative had a single spike followed by a tiny hump (Fig. 11Cc2), and the amperometric response shows two bursts of spikes. (Fig. 11Cc3). (Fig. 11Ca3), and the cumulative charge shows two stepwise increases (Fig. 11Cc4) again. All these results support the notion that the kinetic features of the Ca^2+^ signals predictably establish the attributes of the amperometric response. The example of simultaneous recordings of Ca^2+^ signaling and exocytosis, shown in Fig. 11, is a "proof of concept" of the concepts presented here.

### A possible physiological explanation for one or two Ca^2+^ transient components in rat CCs

One may wonder about the possible reason for double *versus* single voltage-gated Ca^2+^ transient components. We have shown that the Ca^2+^ transients due to Ca^2+^ influx occur with a delay of about 1.1 s. In comparison, the Ca^2+^ signal due to release from intracellular stores following caffeine application occurs with a delay of about 5 s [34]. Then, a possible physiological explanation for the presence of single or double Ca^2+^ transient components is the coupling efficiency between plasma membrane Ca^2+^ entry and Ca^2+^ release from the intracellular Ca^2^ stores (CICR). Strong coupling (perhaps due to proximity between the sites of Ca^2+^ entry and the endoplasmic reticulum or the density of RyRs) would give a single Ca^2+^ component comprising an early Ca^2+^ entry signal broadened by the late Ca^2+^ signal due to CICR. Conversely, a weak coupling, perhaps due to a greater diffusion distance between Ca^2+^ entry sites and intracellular Ca^2+^ stores, would give rise to two Ca^2+^ transient components separated by some latency.

Previously, we reported that membrane depolarization triggered [Ca^2+^]i elevations and catecholamine secretion ~ threefold greater in CCs from SHRs than age-matched WKY rats CCs [34], and that ryanodine, a RyRs blocker, caused a drastic reduction (77%) in amperometric cumulative charge, mainly due to a 71% decrease in the number of spikes [34], demonstrating that CICR contribution to catecholamine hypersecretion in SHR CCs is linked to stronger intracellular Ca^2+^ signals. Possible explanations for the enhanced gain of CICR in SHR CCs include 1. Greater Ca^2+^ content in the ER, 2. increased density of RyRs or endoplasmic reticulum (ER) organelles. 3. RyR hypersensitivity to agonists by deficient calmodulin inhibition.

The effects of acute, reversible inhibition of SERCA with cyclopiazonic acid (CPA) on the exocytotic responses and [Ca^2+^]i changes elicited by brief depolarizations in rat and bovine CCs were examined in CCs from normotensive (WKY) and hypertensive (SHR) rats, which differ in the gain of their CICR [34]. Our results demonstrate that CPA application inhibits the voltage-gated exocytosis similarly in SHR and WKY CCs. Ca^2+^ signals in SHR are inhibited significantly more by CPA than in WKY CCs (*p* < 0.001). Despite the greater phylogenetic distance from the most recent common ancestors, CPA alters the CA secretion in rat CCs more similarly to bovine CCs than mouse CC, suggesting that functional [Ca^2+^]c dynamics and exocytosis in these cells are not subjected to a pronounced natural selection in the phylogenetic tree that relates these species. A small fraction of SHR and WKY CCs display voltage-gated Ca^2+^ signals with early and late components. Simultaneous amperometry also shows two bursts of exocytosis in CCs with two Ca^2+^ components. The late Ca^2+^ transient component is abolished with CPA and ryanodine, and triggered by caffeine, suggesting that it represents the contribution of CICR.

Since ryanodine specifically suppressed CA hypersecretion in SHR CCs, it could be hypothesized that similar agents that inhibit RyR function could attenuate hypertension development in this animal model. However, it has been shown that chronic treatment with dantrolene, an agent known to stabilize RyRs in skeletal muscle and used to treat malignant hyperthermia, did not prevent hypertension development in SHR [22]. On the other hand, agents that inhibit the SERCA pump, such as CPA, suppress catecholamine secretion equally in WKY and SHR CCs. Therefore, they are not likely helpful as therapeutic agents for hypertension. The finding that catecholamine hypersecretion of SHR CCs becomes less marked or disappears after recurrent stimulation might be relevant to the possible role of CCs in the physiopathology of hypertension.

## Data Availability

The datasets generated and analyzed during the current study are available from the corresponding author upon reasonable request.

## References

[1] Alejandre-García T, Segura-Chama P, Parada-Parra OJ, Millán-Aldaco D, Hernández-Cruz A (2023). Calcium Imaging and Amperometric Recording in Cultured Chromaffin Cells and Adrenal Slices from Normotensive, Wistar Kyoto Rats and Spontaneously Hypertensive Rats. Methods Mol Biol (Clifton, N.J.).

[2] Alés E, Fuentealba J, García AG, López MG (2005). Depolarization evokes different patterns of calcium signals and exocytosis in bovine and mouse chromaffin cells: the role of mitochondria. Eur J Neurosci.

[3] Alonso MT, Barrero MJ, Michelena P, Carnicero E, Cuchillo I, García AG, García-Sancho J, Montero M, Alvarez J (1999). Ca2+-induced Ca2+ release in chromaffin cells seen from inside the ER with targeted aequorin. J Cell Biol.

[4] Alonso MT, Chamero P, Villalobos C, García-Sancho J (2003). Fura-2 antagonises calcium-induced calcium release. Cell Calcium.

[5] Álvarez YD, Belingheri AV, Perez Bay AE, Javis SE, Tedford HW, Zamponi G, Marengo FD (2013). The immediately releasable pool of mouse chromaffin cell vesicles is coupled to P/Q-type calcium channels via the synaptic protein interaction site. PloS One.

[6] Amos-Landgraf J, Franklin C, Godfrey V, Grieder F, Grimsrud K, Korf I, Lutz C, Magnuson T, Mirochnitchenko O, Patel S, Reinholdt L, Lloyd KCK (2022) The Mutant Mouse Resource and Research Center (MMRRC): the NIH-supported national public repository and distribution archive of mutant mouse models in the USA. Mamm Genome 33:203–212. 10.1007/s00335-021-09894-0 10.1007/s00335-021-09894-0 PMC8314026 34313795

[7] Barry VA, Cheek TR (1994). A caffeine- and ryanodine-sensitive intracellular Ca2+ store can act as a Ca2+ source and a Ca2+ sink in PC12 cells. Biochem J.

[8] Bryda EC (2013). The mighty mouse: the impact of rodents on advances in biomedical research. Mo Med.

[9] Chow RH, Klingauf J, Heinemann C, Zucker RS, Neher E (1996). Mechanisms determining the time course of secretion in neuroendocrine cells. Neuron.

[10] Coupland RE (1989). The natural history of the chromaffin cell–twenty-five years on the beginning. Arch Histol Cytol.

[11] Cuchillo-Ibáñez I, Olivares R, Aldea M, Villarroya M, Arroyo G, Fuentealba J, García AG, Albillos A (2002). Acetylcholine and potassium elicit different patterns of exocytosis in chromaffin cells when the intracellular calcium handling is disturbed. Pflugers Arch.

[12] Edelstein AD, Tsuchida MA, Amodaj N, Pinkard H, Vale RD, Stuurman N (2014). Advanced methods of microscope control using μManager software. J Biol Methods.

[13] Finkel T, Menazza S, Holmström KM, Parks RJ, Liu J, Sun J, Liu J, Pan X, Murphy E (2015). The ins and outs of mitochondrial calcium. Circ Res.

[14] Friel DD, Tsien RW (1992) A caffeine- and ryanodine-sensitive Ca2+ store in bullfrog sympathetic neurones modulates effects of Ca2+ entry on [Ca2+]i. J Physiol 450:217–246. 10.1113/jphysiol.1992.sp019125 10.1113/jphysiol.1992.sp019125 PMC1176120 1432708

[15] García AG, García-De-Diego AM, Gandía L, Borges R, García-Sancho J (2006). Calcium signaling and exocytosis in adrenal chromaffin cells. Physiol Rev.

[16] García AG, Padín F, Fernández-Morales JC, Maroto M, García-Sancho J (2012). Cytosolic organelles shape calcium signals and exo-endocytotic responses of chromaffin cells. Cell Calcium.

[17] Giovannucci DR, Hlubek MD, Stuenkel EL (1999). Mitochondria regulate the Ca(2+)-exocytosis relationship of bovine adrenal chromaffin cells. J Neurosci.

[18] Hernández-Cruz A (2021). Reversible interruption of ER Ca^2+^ uptake inversely affects ACh-elicited exocytosis in mouse and bovine chromaffin cells. Pflugers Arch.

[19] Hernández-Cruz A, Escobar AL, Jiménez N (1997). Ca(2+)-induced Ca2+ release phenomena in mammalian sympathetic neurons are critically dependent on the rate of rise of trigger Ca2+. J Gen Physiol.

[20] Kumar S, Suleski M, Craig JM, Kasprowicz AE, Sanderford M, Li M, Stecher G, Hedges SB (2022) TimeTree 5: an expanded resource for species divergence times. Mol Biol Evol 39(8), msac174. Advance online publication. 10.1093/molbev/msac174 10.1093/molbev/msac174 PMC9400175 35932227

[21] Lee RM, Borkowski KR, Leenen FH, Tsoporis J, Coughlin M (1991). Combined effect of neonatal sympathectomy and adrenal demedullation on blood pressure and vascular changes in spontaneously hypertensive rats. Circ Res.

[22] Lee JS, Greco L, Migirov A, Li Y, Gerdes AM, Zhang Y (2020). Chronic dantrolene treatment does not affect hypertension, but attenuates sympathetic stimulation enhanced atrial fibrillation inducibility in SHR. Am J Hypertens.

[23] Lovell PV, James DG, McCobb DP (2000). Bovine versus rat adrenal chromaffin cells: big differences in BK potassium channel properties. J Neurophysiol.

[24] Marengo FD, Cárdenas AM (2018). How does the stimulus define exocytosis in adrenal chromaffin cells?. Pflugers Arch.

[25] Martínez-Ramírez C, Gil-Gómez I, de Diego AMG, García AG (2021). Acute reversible SERCA blockade facilitates or blocks exocytosis, respectively in mouse or bovine chromaffin cells. Pflugers Arch.

[26] Miranda-Ferreira R, de Pascual R, Smaili SS, Caricati-Neto A, Gandía L, García AG, Jurkiewicz A (2010). Greater cytosolic and mitochondrial calcium transients in adrenal medullary slices of hypertensive, compared with normotensive rats. Eur J Pharmacol.

[27] Okamoto K, Aoki K (1963). Development of a strain of spontaneously hypertensive rats. Jpn Circ J.

[28] Peña Del Castillo JG, Segura-Chama P, Rincón-Heredia R, Millán-Aldaco D, Giménez-Molina Y, Villanueva J, Gutiérrez LM, Hernández-Cruz A (2021). Development of the hypersecretory phenotype in the population of adrenal chromaffin cells from prehypertensive SHRs. Pflugers Arch.

[29] Raffaello A, Mammucari C, Gherardi G, Rizzuto R (2016). Calcium at the center of cell signaling: interplay between endoplasmic reticulum, mitochondria, and lysosomes. Trends Biochem Sci.

[30] Rigual R, Montero M, Rico AJ, Prieto-Lloret J, Alonso MT, Alvarez J (2002). Modulation of secretion by the endoplasmic reticulum in mouse chromaffin cells. Eur J Neurosci.

[31] Romero-Garcia S, Prado-Garcia H (2019). Mitochondrial calcium: Transport and modulation of cellular processes in homeostasis and cancer (Review). Int J Oncol.

[32] Segura F, Gomez BM, Machado J, Borges R (2000). Automatic analysis for amperometrical recordings of exocytosis. J Neurosci Methods.

[33] Segura-Chama P, Hernández A, Jiménez-Pérez N, Alejandre-García T, Rivera-Cerecedo CV, Hernández-Guijo J, Hernández-Cruz A (2010). Comparison of Ca^2+^ currents of chromaffin cells from normotensive Wistar Kyoto and spontaneously hypertensive rats. Cell Mol Neurobiol.

[34] Segura-Chama P, López-Bistrain P, Pérez-Armendáriz EM, Jiménez-Pérez N, Millán-Aldaco D, Hernández-Cruz A (2015). Enhanced Ca(2+)-induced Ca(2+) release from intracellular stores contributes to catecholamine hypersecretion in adrenal chromaffin cells from spontaneously hypertensive rats. Pflugers Arch.

[35] Stevens DR, Schirra C, Becherer U, Rettig J (2011). Vesicle pools: lessons from adrenal chromaffin cells. Front Synaptic Neurosci.

[36] Voets T, Neher E, Moser T (1999). Mechanisms underlying phasic and sustained secretion in chromaffin cells from mouse adrenal slices. Neuron.

[37] Wightman RM, Jankowski JA, Kennedy RT, Kawagoe KT, Schroeder TJ, Leszczyszyn DJ, Near JA, Diliberto EJ, Viveros OH (1991). Temporally resolved catecholamine spikes correspond to single vesicle release from individual chromaffin cells. Proc Natl Acad Sci U S A.

[38] Wu PC, Fann MJ, Kao LS (2010). Characterization of Ca2+ signaling pathways in mouse adrenal medullary chromaffin cells. J Neurochem.

[39] Yagil Y, Yagil C (2001). Genetic models of hypertension in experimental animals. Exp Nephrol.

[40] Zaika OL, Pochynyuk OM, Kostyuk PG, Yavorskaya EN, Lukyanetz EA (2004). Acetylcholine-induced calcium signalling in adrenaline- and noradrenaline-containing adrenal chromaffin cells. Arch Biochem Biophys.

